# A Scoping Review of the Health of African Immigrant and Refugee Children

**DOI:** 10.3390/ijerph18073514

**Published:** 2021-03-28

**Authors:** Bukola Salami, Higinio Fernandez-Sanchez, Christa Fouche, Catrin Evans, Lindiwe Sibeko, Mia Tulli, Ashley Bulaong, Stephen Owusu Kwankye, Mary Ani-Amponsah, Philomina Okeke-Ihejirika, Hayat Gommaa, Kafuli Agbemenu, Chizoma Millicent Ndikom, Solina Richter

**Affiliations:** 1 Faculty of Nursing, University of Alberta, 11405 87 Avenue, Edmonton, AB T6G 0Z7, Canada; higinio@ualberta.ca (H.F.-S.); tulli@ualberta.ca (M.T.); abulaong@ualberta.ca (A.B.); solina.richter@ualberta.ca (S.R.); 2 Faculty of Education and Social Work, University of Auckland, Private Bag 92019, Auckland 1142, New Zealand; c.fouche@auckland.ac.nz; 3 School of Health Sciences, University of Nottingham, University Park, Nottingham NG7 2RD, UK; Catrin.Evans@nottingham.ac.uk; 4 Department of Nutrition, School of Public Health and Health Sciences, University of Massachusetts, 204 Chenoweth Laboratory, Amherst, MA 01003-9282, USA; lsibeko@umass.edu; 5 Regional Institute for Population Studies, University of Ghana, P.O. Box LG 96, Legon, Accra GA184, Ghana; skwankye@ug.edu.gh; 6 School of Nursing, University of Ghana, P.O. Box LG 43, Legon, Accra GA184, Ghana; mnkansah@ualberta.ca; 7 Department of Women’s and Gender Studies, University of Alberta, Edmonton, AB T6G 2E7, Canada; pokeke@ualberta.ca; 8 Department of Nursing Science, Ahmadu Bello University, Sokoto Road, PMB 06, Zaria 810107, Nigeria; h_gommaa@yahoo.com; 9 School of Nursing, The State University of New York (SUNY), University at Buffalo, 3435 Main Street, Buffalo, NY 14214, USA; agbemenu@buffalo.edu; 10 Department of Nursing, University of Ibadan, Ibadan 200284, Nigeria; cmndikom@gmail.com

**Keywords:** migration, health, child health, Africa

## Abstract

Migration is a growing phenomenon around the world, including within the African continent. Many migrants, especially African children, face challenges related to health and social inclusion and can face increased health risks. A systematic scoping review of available literature on the health of African migrant children across the globe was conducted to offer insight into these health risks. The review was conducted over a 15-month period from January 2019 to April 2020, yielding 6602 articles once duplicates were removed. This search included electronic databases, reference lists of articles reviewed, and searches of libraries of relevant organisations. A total of 187 studies met the inclusion criteria, of which 159 were quantitative, 22 were qualitative, and 6 used mixed methods. The findings reveal decreased health in this population in areas of nutrition, infectious diseases, mental health, birth outcomes, sexual and reproductive health, physical and developmental health, parasitic infections, oral health, respiratory health, preventative health, endocrine disorders, health care services, and haematological conditions. The findings offer insights into factors influencing the health of African immigrant and refugee children. Further studies, especially qualitative studies, are needed to determine barriers to service access after migration and to investigate other underexplored and overlooked health concerns of African migrant children, including pneumonia and child maltreatment.

## 1. Introduction

Global migration is a growing phenomenon heavily influenced by global economic, social, political, and technological transformations, and climate change [1]. The International Organisation for Migration [1] estimates that of some 272 million international migrants, of which approximately 31 million (13.9%) are children ages 0 to 18 years old. Migration commonly follows a pattern of movement from low- to higher-income countries, including within the African continent, which has a growing number of migrants. In 2019, there were approximately 39.5 million African migrants, of which 21 million moved within African countries and 18.5 million migrated outside of Africa [1]. The vast majority of migrants are labour migrants.

While migrants make significant contributions to the economic, socio-cultural, and political growth of their destination countries, they also face challenges related to health and social inclusion. Migration can lead to increased health risks, but also potentially to improved health for those who seek safety, including children [1]. Differences and complexities are related to the health of migrants, including issues that cross racial, ethnic, and generational lines. While research has identified a health advantage for economic immigrants, compared to the domestic population of host countries (a phenomenon known as the healthy immigrant effect), the case is not the same for refugees who flee their countries for safety [2,3,4]. Research also points to poorer mental health among second-generation immigrant children, compared to first-generation immigrants [5,6]. First-generation immigrant children have been shown to demonstrate better mental health than non-immigrant children [5], which is evidence of the healthy immigrant effect [6]. However, favourable health status among immigrants may be due to the fact that previous research has often combined all immigrant children without considering the differences among them. For instance, immigrant children and youth from certain regions of Africa, especially those who are refugees, have poorer mental health status than non-immigrant children [7]. The distinct health vulnerabilities faced by African immigrant children create a need to address the mental health of this population. This paper presents the results of a scoping review of the literature on the health of African migrant children (AMC).

Health is a human right that can influence the ability to enjoy other rights. However, many African migrants experience health challenges upon settlement in a new country [8,9]. Pre-migration factors (e.g., experience of trauma) and post-migration challenges (e.g., access to health care) contribute to the health of immigrants in destination countries [1,10]. A scoping review that included 14 articles found that culture, religious beliefs, linguistic barriers, and barriers accessing health care contributed to the health of African immigrants in the United States [8]. Barriers to access to health care for this population include cost, health system complexity, lack of culturally competent health providers, long wait times, and discrimination [8,11]. Moreover, despite an overall higher level of education than native-born populations and other immigrant groups, African migrants to high-income countries often experience a higher rate of poverty, a key social determinant of health [12]. Evidence suggests that income is a major contributor to immigrant health [13]. As is the case with other marginalised groups, social determinants of health play a critical role in the decline of immigrant health after a period of time in the destination country [13], and this is especially true for African immigrants [9]. The poor social outcomes of immigrants extend to the health of their children, which is particularly the case for African migrants who often have to juggle multiple jobs to earn the income necessary to meet the health needs of their children [14]. Poor social outcomes of immigrant parents impose intergenerational consequences, whereby second-generation immigrant children experience poorer health outcomes than their parents [15,16]; again, this is most evident in the case of African immigrants.

Immigrant and refugee children experience several health challenges upon arrival in their destination countries. Despite the fact that these children’s health should be protected as a human right, overwhelming evidence from systematic reviews indicates refugee children experience poor mental health status, especially post-traumatic stress [17,18,19]. Pre-migration experience and post-migration stress contribute to the mental health of refugee children [17]. Social support, sense of belonging, and connection to home culture can improve resilience in this population and contribute to improved mental health [20]. Immigrant and refugee children also experience physical health problems, including anaemia, haemoglobinopathies, chronic hepatitis B, latent tuberculosis infection, intestinal infection, oral health problems, vitamin D deficiency, wasting, and stunting [21,22]. While several systematic reviews have been published on the health of immigrant and refugee children, we found none that focus on the health of African immigrant children; previous reviews often combined African immigrant children with other immigrants and lacked an in-depth analysis of their particular vulnerabilities. Thus, a review of existing studies is necessary to fill this gap. A review focusing on African immigrant children’s health is particularly important given the poor health status of African immigrants [7,8]. Further study of the health status of African immigrants is also needed to ensure and inform the development of relevant interventions. Interventions should involve the participation of, and centre the voices of, African migrant and refugee children.

## 2. Materials and Methods

A scoping review is appropriate for identifying existing research activity on a particular topic and key research gaps [23,24]. We conducted a systematic scoping review over a 15-month period from January 2019 to April 2020. This review followed the five-stage approach to scoping reviews as outlined by Arksey and O’Malley [25]: developing the research question; identifying the relevant studies; selecting the articles; data charting and data extraction; and collating, summarizing, and reporting the results. The review has been reported following the Preferred Reporting Items for Systematic Reviews and Meta-Analyses (PRISMA), Extension for Scoping Review Guidelines [26].

Stage 1 aimed to refine questions on the body of evidence related to African immigrant and refugee children. Our research questions were as follows:

1. What is the scope, range, and nature of evidence on the health of African migrant children?
2. What is known from the existing literature on the health of African migrant children?

Stage 2 involved identifying the relevant studies. The search included electronic databases, a reference list of articles reviewed, and searches of libraries of relevant organisations. The following databases were searched on 21 January 2019 by a health science librarian: Medline, Embase, Ovid Global Health, Ovid PsycInfo, Cochrane Library, CINAHL, SocIndex; EBSCO Child Development and Adolescent Studies, ProQuest Sociological Abstracts, and ProQuest Dissertations and Theses Global. The search terms used represent associated keyword combinations of child health, immigrant health, and Africa (Table 1: Search Terms). A total of 12,720 records were identified and retrieved, and 6602 records remained after duplicates were removed. These articles were exported into Covidence, an online software that works in collaboration with Cochrane to enhance the selection and completion of systematic reviews. This software allows for independent reviewers to screen records based on inclusion criteria. Results were checked by two research assistants.

**Figure 1:**
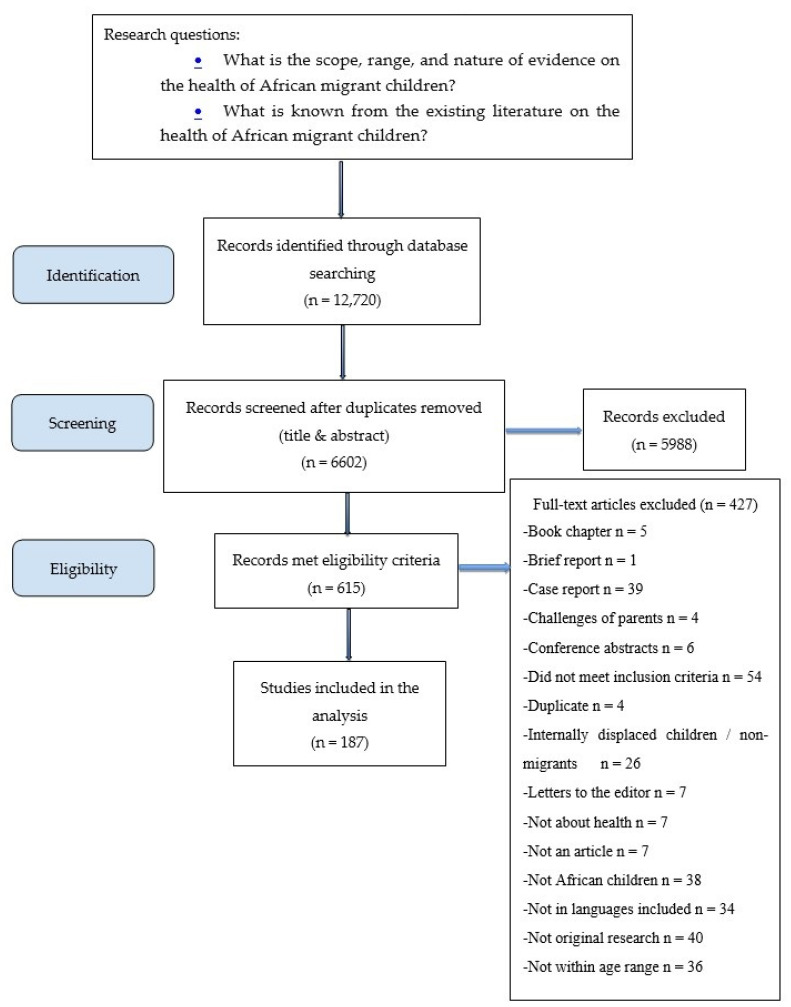
Flow Chart.

Stage 3 involved article selection. Two research assistants independently selected and reviewed each article that met the following inclusion criteria: (1) focus on the health of African child migrants and refugees; (2) child’s age is between 0 and 18 years old; (3) child is either a first-generation or second-generation immigrant; (4) report original research studies; and (5) data are on children who crossed an international border. We excluded articles related to internally displaced children and internal migrants because they are still located within the same country and policy jurisdiction. We also excluded articles on African American children (in which information was not provided on whether they are immigrants), African Caribbean children, and articles that focused on the challenges of parents without sufficient information on the health of the child. We also excluded articles that did not have disaggregated data on African immigrant children (e.g., those in which data on African immigrants were combined with other immigrants). All publications such as conference abstracts, literature reviews, book chapters, brief reports or case reports, posters, study protocols, and theses that did not qualify as an empirical output were also excluded (see list in the flow chart). Screening of titles and abstracts and excluding articles resulted in a remaining 615 articles. Only articles published in English and Spanish were included. We screened the full text of all 615 and rejected another 426 based on the inclusion criteria to arrive at 187 articles used in the analysis (see Figure 1 for PRISMA flow chart). At each stage, disagreements between the two reviewers were resolved by consensus or by a third reviewer.

Stage 4 involved data charting and data extraction. The following information was extracted from each of the 187 articles: author(s) name, year of publication, research questions or objectives, theoretical framework, method (sampling, sample size, age of the child, data source (parent vs. child vs. health professional vs. medical records vs. clinical assessment vs. laboratory tests), clinical area of focus (e.g., mental health, nutrition, cardiovascular health), period of data collection, country of origin or region, destination country or region, and summary of findings. In total, six research assistants extracted and collated data. Each paper was reviewed by one research assistant and verified by another for accuracy. The research assistants received four hours of training on scoping reviews and attended weekly meetings to discuss emerging findings.

Stage 5 involved collating, summarizing, and reporting the results. Given the vast number of articles included, we coded and summarised data based on areas of focus and thematically analysed the content. We also compared data across the coding segments. The purpose of the comparison was to identify relevant linkages (relationships, interactions, and consequences) across data. Numerical data are represented using descriptive statistics, frequencies, measures of central tendency, and measures of variability. As a scoping review, the purpose is to map out the existing evidence and summarise key study findings within different domains rather than evaluate the quality of individual studies to determine the risk of bias related to particular outcomes. Hence, in line with the review aim of providing an overview of the existing evidence base, a formal quality assessment of each study was not undertaken [25].

## 3. Results

### 3.1. Characteristics of Included Studies

A total of 187 studies were ultimately included in this scoping review (Figure 1). Supplementary Table S1 shows the key characteristics of the articles. The majority of the studies were quantitative (n = 159), with less representation in terms of qualitative (n = 22) and mixed methods (n = 6) research. The oldest year of publication was 1965, and the most recent article was published in 2019. The majority of studies either did not mention their sampling method (n = 133) or utilised a non-probabilistic method (n = 42). Data were collected from diverse sources including children, parents, medical records, national registries, and health care professionals. The top three modes of data collection were via multiple methods (n = 63), surveys and questionnaires (n = 38), and diagnostic or laboratory testing (n = 34). Few studies reported the use of a theoretical framework (n = 10); those that did, used frameworks based on health services for community living, cognitive theory, a social-ecological framework, a cultural competence framework, the health access model and household resources model, anthropological theory, the theory of segmental assimilation, narrative inquiry and ethnographic impressionism, and transgenerational trauma transference.

### 3.2. Characteristics of Study Participants

A broad range of children’s ages was examined: under 1 year old (n = 25), 1–6 years old (n = 30), 7–11 years old (n = 22), and 12–18 years old (n = 133). Most studies either reported on migrants from multiple countries (n = 37) or generalised to migrants from Africa (n = 28). Others reported participants were from Somalia (n = 27), Ethiopia (n = 24), and sub-Saharan Africa (n = 13). European (n = 69) and African (n = 39) countries were the primary destinations, although a notable number of studies were conducted in Israel (n = 26), the United States (n = 22), and Australia (n = 21).

### 3.3. Identified Themes

The studies included in this review report on 11 areas of health, namely, infectious diseases (n = 25), including parasitic infections (n = 17); nutrition (n = 40); mental health (n = 28); birth outcomes and sexual/reproductive health (n = 23); physical and developmental health (n = 19); oral health (n = 16); respiratory health (n = 9); preventative health (n = 8); endocrine health (n = 8); health care services (n = 8); and haematological conditions (n = 5).

#### 3.3.1. Infectious Diseases, Including Parasitic Infections

Overall, 25 of the articles focus on infectious diseases [27,28,29,30,31,32,33,34,35,36,37,38,39,40,41,42,43,44,45,46,47,48,49,50,51]. The literature indicates high rates of human immunodeficiency virus (HIV) infection in AMC [45,46,47,48,49,50], and Germany [51]. Studies in Israel and Belgium found a high prevalence of mother-to-child HIV transmissions [45,48]. AMC living with HIV in the United States and the Netherlands are often diagnosed in the host country, with antiretroviral therapy started at an older age [47,50] However, HIV-infected parents migrating to Sweden are unlikely to disclose their status to their children due to fear of double stigma [46].

Research shows hepatitis A and B are prevalent among AMC in Italy and Spain [27,28]. Similarly, problems with *Chlamydia trachomatis*, cholera, and varicella-zoster virus are evident in AMC in the United States and Algeria [29,30,31]. The presence of measles and rubella also affects the health of AMC in Cameroon and Cote d’Ivoire [32,33], as did *Heliobacter pylori* in Australia, although the latter was reduced by premigration antimalarial therapy [34,35,36,37]. Tinea capitis is a common infectious disease among AMC in New Zealand [38], Israel [39], Spain [40,41], and Australia [42]. Other scalp infections from *Trichophyton violaceum* and *Trishophyton soudanense* are also present in AMC living in Sweden and the United States [43,44].

A total of 16 articles report on parasitology—seven report on malaria [52,53,54,55,56,57,58], four discuss helminth infections [35,36,37,59], and five focus on unspecified infections [60,61,62,63,64]. Intestinal parasites are common among AMC in the United States, Spain, and Australia [61,62,63,65]. Similarly, the infestation rate of head lice among AMC in Israel is high [64]. Among migrants to Spain, parasitic infections are most common in people originating from sub-Saharan and North Africa and are frequently associated with eosinophilia [62]. However, AMC in England have much lower helminth carriage rates than other migrant populations [59]. In Australia, helminth infections are also common but are not associated with reduced growth or with *H. pylori* infection [36], are not predicted by gastrointestinal symptoms and significantly elevated cytokines [37], and do not elevate urinary hepcidin [36]. The literature also shows imported childhood malaria is increasing among AMC in England [58] and Spain [52]. Similarly, *Plasmodium falciparum* infections are disproportionately high among AMC compared to populations in receiving countries such as Canada [53], the Democratic Republic of Congo [54], Australia [55], and Zambia [56]. Prevalence is thought to be partly connected to malnutrition [54], and receiving countries must be prepared to treat children for *P. falciparum* infections on arrival.

#### 3.3.2. Nutrition

A total of 40 articles report on nutrition following arrival to the host country [65,66,67,68,69,70,71,72,73,74,75,76,77,78,79,80,81,82,83,84,85,86,87,88,89,90,91,92,93,94,95,96,97,98,99,100,101,102,103,104]. Concerning breastfeeding practices among mothers of AMC, scholars largely centre their attention on high-income settings such as Norway and Australia, or conversely on refugee camps in Africa. In developed countries, some authors report positive attitudes towards breastfeeding [66]; however, others note an interruption of breastfeeding at six months post-birth primarily due to insufficient milk supply [67], a decline in breastfeeding after arrival to the host country [68], and increased challenges to exclusive breastfeeding [69]. In contrast, refugees in Ghana improved their breastfeeding practices with increasing time in the receiving country [70]. Infant refugees to Algeria often experience excessive iodine intake [71] and are constantly underweight [72].

Evidence on dietary choices and feeding behaviours of AMC vary across settings. In Algeria, Italy, and Spain, several authors report lower quality diets in AMC soon after their arrival [73,74]. For instance, some immigrants to Italy are less likely to have breakfast compared to Italians [75]. Conversely, AMC in Norway appear to align with Norwegian dietary recommendations [76], and AMC in the United States make dietary choices that are highly influenced by their culture [77]. Factors associated with child hunger and food insecurity include poor English proficiency and shorter length of stay in the host country [78,79].

Issues related to obesity and overweight have appeared upon resettlement of AMC in host countries including the United States, the Netherlands, and Australia [80,81,82,83]. Some authors attribute these health issues to cigarette smoking [84] and changes to traditional lifestyle [81]. Similarly, vitamin D deficiency or insufficiency is a frequent concern for AMC living in Australia and Germany [85,86,87]. In contrast, AMC with body mass indexes (BMIs) indicating underweight problems are also reported in the literature, specifically in Canada [88], Saudi Arabia [89], Zaire [90], Israel [91], Kenya [92], Scotland [60], the United States [65], and in migrants to Kenya, Ethiopia, Uganda, and Algeria [93]. Some authors credit weight issues to non-consumption of milk, low literacy of fathers, and having no immunisation card [94].

To resolve nutrition-related concerns of AMC, host countries such as Australia have designed, implemented, and evaluated culturally competent obesity-prevention programs through community-partnered participatory approaches [95]. Likewise, lipid-based nutrient supplement programs have been implemented in Algeria [96], nutrient-dense fortified spread programs in the United States [97], and maize meal fortification in Zambia [98]. However, despite an increase of interventions, acute malnutrition remained prevalent in Somalia [99], no significant change in haemoglobin with micronutrient powder was observed in Kenya [100], and malnutrition rates for migrants to Zaire persisted [101].

#### 3.3.3. Mental Health

In total, 25 of the articles focus on the mental health of AMC [105,106,107,108,109,110,111,112,113,114,115,116,117,118,119,120,121,122,123,124,125,126,127,128,129,130,131,132]. Post-traumatic stress disorder (PTSD) is linked to having experienced traumatic events such as war and violence [105,106,107]. Girls are more likely to develop this problem [107]. Other mental health issues among AMC in Israel include suicide ideation and alcohol abuse [108,109], anxiety, and depression [109,110]. Similarly, in Uganda, exposure to violence is associated with higher symptoms of depression among AMC [111], and the psychosocial well-being of AMC is impacted by the potential influx of refugees [112]. However, in Norway, AMC experience less stressful events compared to migrants from Asia [113], and in Australia, the social-emotional well-being of AMC improves over time, with children who had four or more protective factors at low risk of poor social-emotional well-being [114].

Many authors report issues of discrimination, racism, and emotional breakdowns experienced by AMC in the Netherlands [115], Canada [116], and the United Kingdom [117]. Likewise, behavioural problems are seen to arise with ambivalent acculturation patterns [105,118] and negative perspectives on mental illness in AMC [119]. In Canada and Israel, some authors identify personal characteristics, interpersonal relationships, environmental characteristics, and the family as factors that influence AMC’s identity and sense of belonging [120,121]. In Italy, AMC who integrated into the national culture show higher self-esteem, life satisfaction, and socio-cultural competence relative to their non-integrated counterparts [122]. Despite the mental health concerns among AMC, contact with mental health services in destination countries such as the UK appears limited [123].

Three articles discuss child trafficking among AMC [124,125,126]. Iyakaremye and Mukagatare [124] note that child trafficking is facilitated by the poor standard of living in the refugee camp, convoluted camp layout, and inadequate security system, which negatively impacts girls’ reproductive health, social integration, and mental health. As a result, researchers in Rwanda found that stigma negatively impacted girls’ access to health services [125]. Finally, Warria [126] concludes that cultural-responsive care becomes a highly important practice method for a child welfare practitioner when engaging with transnational trafficked children from diverse sociocultural, linguistic, economic, and ethnic backgrounds.

#### 3.3.4. Birth Outcomes and Reproductive and Sexual Health

Overall, 23 of the articles included in this review examine birth outcomes, with less focus on the reproductive and sexual health of AMC [133,134,135,136,137,138,139,140,141,142,143,144,145,146,147,148,149,150,151,152,153,154,155]. A higher prevalence of preterm births is seen in Portugal and Belgium [133,134,135], while infant mortality is prevalent in places including Tanzania [136], Canada [137], Italy [138], and South Africa [139]. Similarly, perinatal mortality in African migrant women is high in Sweden [140] and Belgium [135]. Birthweights of AMC vary across studies; for instance, some authors report low birthweights or high risk for low birthweights in Belgium, Israel, and Spain [141,142,143,144], whereas others note higher birth weights among AMC in these same countries and less likelihood of being born preterm or with low birth weight [145,146,147]. In the United States, researchers found new-borns to Somali immigrants are at increased risk for prolonged hospitalisation, lower 5-min Apgar scores, assisted ventilation, and meconium aspiration [148]. In contrast, other authors report no relationship between preterm birth and low birth weights [149].

In terms of sexual and reproductive health, in Ethiopia, AMC reported multiple sources of sexual and reproductive health information including parents, peers, and religious leaders [150]. Migrants to Portugal reported difficulties talking about sex with parents, the tendency to begin sexual life early, and the infrequent use of condoms [151]. On the other hand, some authors report no evidence linking female circumcision to perinatal death in migrants to Sweden [152] and confirm the significance of non-therapeutic male circumcision in migrants to Spain [153]. In addition, the literature shows Somali girls accept but are less likely to complete the human papillomavirus (HPV) vaccine series [154], and AMC in the United States who live in minority diversity neighbourhoods saw protective effects on infant health [155].

#### 3.3.5. Physical and Developmental Health

A total of 19 articles discuss physical and developmental health [61,95,119,156,157,158,159,160,161,162,163,164,165,166,167,168,169,170,171] and shed light on AMC’s growth and development. For example, AMC in Israel, the United States, and France are more likely to fail tests assessing fine-motor skill, linguistic, and socio-emotional domains [156], and to have poor growth [61] and a low BMI [61]. In addition, AMC in the USA represent the majority of profound biotinidase deficiency (BTD) cases; however, none had partial BTD [157]. Conversely, AMC in Scotland grow to taller heights than Asian migrants [158].

Ethiopian child migrants to Israel have lower rates of pervasive developmental disorder (PDD), compared to immigrants from other countries and native Israeli children [159]. AMC in Sweden and the United States have a high prevalence of autism spectrum disorder (ASD) [160,161,162]. In other receiving countries, some authors report familial challenges to the acceptance and understanding of child migrants’ autism, which include cultural attitudes towards mental illness, behaviour, and disability, in addition to a lack of vocabulary to describe the disorder [163]. Additionally, AMC in Australia report a negative perspective on mental illness within the community, with 85% reporting bad perceptions of ASD [119].

AMC in the Netherlands are significantly more likely to watch television for two or more hours per day, compared to native Dutch children [164]. East African migrants report a desire to be physically active, but migrants to both the United States and Australia face challenges to physical inactivity, including social, environmental, and cultural barriers [165,166]. On the contrary, Somali child migrants to the United States are more likely to play soccer than other black peers; the girls have lower activity levels compared to black and white peers, but no differences are noted for boys [167].

Non-communicable diseases such as neonatal jaundice [168] sickle-cell disease [169], and Kawasaki disease [170] are common in AMC in Norway, Canada, and the United States.

#### 3.3.6. Oral Health

In total, 17 of the included studies focus on oral health [61,65,172,173,174,175,176,177,178,179,180,181,182,183,184,185], with many examining the oral health of AMC in Israel. While some report an issue with missing teeth [172,173], others note low levels of caries, especially if the AMC has recently arrived [65,174,175,176,177]. Additionally, parents of AMC report less frequent daily toothbrushing and dentist visits, when compared to their Israeli counterparts [178]. Few or no dental visits are common among AMC in the United States [184] and Canada [179]. Dental abnormalities, poor oral hygiene, and prevalence of caries are also common among AMC [61,181] Barriers to the prevention of early childhood caries are associated with home-based prevention, early detection, and health care access [182].

#### 3.3.7. Respiratory Health

Nine articles discuss respiratory health [61,186,187,188,189,190,191,192,193]. AMC in Israel, the United States, and the Netherlands report issues with asthma; however, most show a lower prevalence and better health-related quality of life, compared to local children [186,187,188]. Authors report issues with tuberculosis (TB) among AMC in the United States, Sweden, Israel, and Australia [61,189,190,191,192,193]. The appearance of this disease has been linked to lower height-for-age z scores [61]. AMC are typically diagnosed within three years upon arriving in the host country [190], and asthma in young AMC (0–4 years) is frequently detected [190].

#### 3.3.8. Preventative Health

Nine studies report on preventative health in AMC [30,154,156,194,195,196,197,198,199]. Paxton et al. [194] note that 97% of AMC have incomplete or uncertain immunisation status, while other authors suggest serological screening to update the immunisation status of AMC in Italy [195]. In Malawi, evidence shows AMC sometimes lack their second dose of measles vaccine [196]. Similarly, in the United States, girls generally accept initiating the HPV vaccine but less likely to complete the series [154]. In contrast, AMC in Israel and the United States have complete vaccination schedules [154], and their antibody levels against many viruses are similar or higher than non-immigrants [197]. Regarding cardiovascular health, Colombatti et al. [198] declare a need for parental education in the native language on stroke risk and prevention of sickle cell disease (SCD) to increase compliance. Likewise, two studies point out the need for updated policies, education, and an increase of vaccine uptake or routine testing for antibodies [30,199].

#### 3.3.9. Endocrine Health

Of the eight articles that report on endocrine health, four focus on type 1 diabetes (T1D) [200,201,202,203] and four on thyroid pathologies [71,204,205]. The literature shows T1D is often present in AMC in host countries such as Italy, the Netherlands, and Sweden [200,201,202], and that this issue often develops after arrival in host countries [200]. Further, AMC have a higher risk of T1D with increasing time their mother spent in the receiving nation [201]. In contrast, AMC in Finland have a similar prevalence of T1D as Finnish children [203].

On the other hand, researchers report excessive iodine intake in infants in Algeria [204] and children suffering from endemic goitre likely caused by excessive iodine intake [205]; a relationship between excessive iodine intake with thyroid dysfunction and poorer developmental status is reported [204]. Conversely, Ethiopian immigrants to Israel experience a high prevalence of goitre with a low frequency of hypothyroidism, which may be attributed to food goitrogens and iodine deficiency prevailing in Ethiopia [206].

#### 3.3.10. Health care Services

Eight of the studies included in this review analyse health care services [119,123,207,208,209,210,211,212]. AMC in the UK indicate not having contact with health care services [123]; AMC in Israel adopt Western practices and go to the clinic for medical treatment, but if this does not prove satisfactory, they seek out solutions according to familiar patterns from their country of origin for medical, social, or emotional problems [207]. Similarly, AMC caregivers in South Africa report dissatisfaction with health care services [208], and AMC in Spain report a sense of pointlessness in seeking out health care services [209]. On the contrary, AMC in Australia have highly accessible primary care [119,210], and the major barriers to access are the availability of interpreters and lack of information [211]. For migrants to Uganda, proximity, maternal perceived severity of illness, financial resources, and permission from the father determine treatment access [212].

#### 3.3.11. Haematological Conditions

Five of the included articles focus on haematology concerns [158,169,213,214,215]. For instance, Plotinsky et al. (2008) [213] note that blood lead levels (BLLs) of refugees are significantly higher than among non-refugee children. Similarly, Goel et al. (2009) [158] suggest nutritional iron deficiency remains the most common cause of anaemia in African immigrants. AMC in Canada represent 25% of all patients with SCD born abroad [214]: some authors suggest that early recognition and intervention may reduce this problem [215]. In addition, bilingual migrant children to Italy with SCD display a rate of cognitive impairment similar to their monolingual counterparts but have a more pronounced and precocious onset of language difficulties [169].

## 4. Discussion

In providing a comprehensive overview of the empirical evidence on AMC, this scoping review systematically synthesises the breadth, range, and nature of the evidence on the health of African migrant children across the globe. It is crucial to scope the literature on African migrant children to map the research in the field and gain more insight into the gaps in the literature. Due to our large time range (1967–2019), it is important to note that changes in policies over time may affect migrants. Our findings indicate that scholars have largely focused on the nutritional, mental, and infectious diseases of migrant children from Sudan, Ethiopia, and Somalia who are living in European countries such as Spain and Italy. Results from our review indicate most included articles utilise a quantitative approach, with only a few incorporating a theoretical lens to guide research. More qualitative and participatory action research studies are needed because these approaches are essential to amplify the voices of African migrant children further through research processes. While many of the studies included children as data sources, these were overwhelmingly quantitative. Only 10 studies were qualitative and gathered data from migrant and refugee children from countries in sub-Saharan Africa [112,116,120,124,125,129,132,150,166,207]. The rest of the qualitative studies gathered data about children from parents or service provider participants. In addition, there is a need to focus on children as bearers of rights under the United Nations Convention on the Rights of the Child. For example, children have the right to have basic needs met and the right to access the necessities of a good life. Based on these discoveries, we make recommendations for practice, public health policy, and future research.

Evidence on African immigrants and refugees in a mental health care setting shows that, by actively involving study participants, researchers are able to ensure meaningful and culturally appropriate scholarly work [216]. Similarly, Murray et al. [217] found participant engagement in the research process positively influenced physical activity behaviour in East African communities in the United States; these engagements undoubtedly tap into communal agency, knowledge, and sense of belonging. Worth noting also is that many articles included in this review lack a theoretical framework. We, similar to others, argue that theory can permeate the entire research process, thus allowing intellectuals to enhance the plan, design, and evaluation of their investigations [218,219,220]. We suggest a theory-driven approach can add value to future research on AMC, including intervention work.

In 2017, of the 36 million African transnational migrants, 19 million live within the African continent, and the rest migrated transcontinentally; African migrants younger than 24 years of age made up 38% of the total number of migrants, over six million were migrant children, and about seven million were internally displaced [221]. Data from 2019 show African migrants and refugees continue to move within the African continent, with Egypt, Morocco, Algeria, and Sudan among the top sending countries and Cote d’Ivoire and South Africa the leading host countries [1]. Even though European countries continue to be the top destinations for African migrants who go pancontinental, other countries, such as Canada, have recently reported an arrival increase of African nationals [222]. Thus, further research is needed in countries that host large numbers of AMC, such as South Africa and Canada. Again, contrary to the long-publicised wave of African migrants moving away from the continent in the media, further research on African migrants within the continent itself is also needed because, as observed in this review, a relatively higher proportion of African migrants continue to be hosted in other African destination countries. In West Africa, for example, there is considerably more interaction among countries within the sub-region due to the Economic Community of West African States (ECOWAS) free movement of people and goods protocol. Quite admittedly, however, destination countries outside the African continent are more unfriendly and unfamiliar for many migrants compared to those within Africa and, therefore, the health-related outcomes for many AMC outside the African continent may be graver than for those within the region but outside their countries of origin. Finally, the current global COVID-19 outbreak will undoubtedly have impacts on the health of AMC. Future studies are needed to understand these impacts and implications.

The results from this scoping review underline major issues that could be related to the health beliefs and cultural practices of migrant families that impact child health. Many scholars report the poor communication between AMC and their parents about sex, a lack of importance given to their oral health and dietary choices, and inadequacy of breastfeeding practices. These findings are consistent with other research on African immigrant populations, in which scientists have found traditional beliefs of African immigrant women interfere with exclusive breastfeeding during the first six months of a child’s development [223]. Likewise, African immigrants in college culturally perceive American food as unhealthy, yet they become acculturated to it [224]. In contrast, Cooper Brathwaite and Lemonde [225] discovered that immigrants from Ghana and Nigeria rely on their health beliefs and cultural practices in the prevention of type 2 diabetes, and also preserve their culture through their food choices.

The articles included in this scoping review report on the experiences of AMC and the health care system. Studies show barriers across a range of destination country contexts include language barriers and a lack of translation services [226], long wait times and dissatisfaction with care [11,208], and financial costs, especially concerning non-basic health care [11]. As explained above, migration and settlement journeys can impact health and access to care in new countries. Lack of status or proper documentation is an acute barrier to access. For example, even though children under six years of age in South Africa are entitled to health care regardless of status, they often do not have access to the necessary paperwork [227].

Our results echo other research with immigrant populations, in which discrimination, racism, fear, cultural diversities, and the lack of effective communication with health care providers often lead to dissatisfaction towards health care services [228,229,230]. These barriers to African immigrant and refugee children’s access to health care have demonstrable impacts on child health. Data from the United States show race and immigration status impact youth health in intersectional ways; specifically, many Somali immigrants and refugee children are not meeting Healthy People 2020 Objectives, scoring particularly low in mental health and healthy weight goals [231]. There is an urgent need for culturally trained practitioners, especially in areas high in migrants. Cultural competency interventions have been shown to improve a series of outcomes such as access to health care and patient outcomes [232]. Hence, we suggest the implementation of these interventions could help to improve the satisfaction of AMC with the health care systems in host countries by increasing awareness of cultural norms among health care providers.

Finally, childhood tuberculosis and latent tuberculosis are important topics to consider in African Immigrant children’s health. However, this scoping review found little data concerning either diagnosis and treatment or prevention among this population. More studies are needed to fill this gap.

## 5. Conclusions

Our findings suggest a need to investigate other least researched health concerns of AMC. The World Health Organization [233] reported that, in 2015, pneumonia killed more than 900,000 African children under 5 years old, and about a quarter of all African adults had suffered physical violence during their childhood. Similarly, AMC to Europe are repeatedly exposed to maltreatment during their migration journey [234]. Child maltreatment can include physical, emotional, or sexual abuse, neglect, negligence, or exploitation. In this regard, we suggest the creation of policies aimed at providing health assessments for AMC to identify individualised health care needs as soon as possible. Health examinations should emphasise the newly arrived AMC and also involve continuous care. This is important because evidence shows recent immigrants present a healthy immigrant effect [235]. As UN Member States aim to achieve universal health coverage (UHC) by 2030, which is a critical part of the Sustainable Development Goals, it is imperative to optimise full access to the range of essential, quality health services, from health promotion to preventive, curative, and rehabilitative care. 

## 6. Limitations

This scoping review provides a comprehensive overview of the empirical evidence on AMC; however, it has its limitations. Due to the nature of the methodology, details of the included studies are not discussed in this article, nor is a formal critical appraisal or bias assessment available. Although we conducted a broad search, some articles might have been missed if they were published in other languages, used alternative keywords, were published in African journals not indexed in the databases searched, cultural sensitivity in service provision, climatic adaptation, and clothing support. Additionally, there is a limitation in considering only the experiences of sub-Saharan migrants and excluding those from North Africa. However, we make this distinction due to differences in culture between countries in North Africa and other regions.

## Figures and Tables

**Table 1:** Search Terms.

	Keywords Used
Child Health	Adolescent health services, child care, infant care, infant, newborn, infant health, child welfare, infant welfare, child nutrition disorders, infant nutrition disorders, child nutritional physiological phenomena, adolescent nutritional physiological phenomena, adolescent development, adolescent health, adolescent medicine, adolescent psychiatry, psychology, adolescent or child psychiatry, adolescent development, child development, psychology, child, children, childhood, infant *, newborn *, neonate *, baby, babies, preschool *, toddler *, adolescen *, teen *, youth *, pediatric *, paediatric *
Immigrant	Emigrants, refugee *, immigra *, asylum seeker *, migrant *, displace *, transient *
Africa	African * and the name of each African country

* denotes all variations of the term.

## Supplementary Materials

The following are available online at https://www.mdpi.com/article/10.3390/ijerph18073514/s1, Table S1: Key Findings.
