# Supplementary material for: A Scoping Review of the Health of African Immigrant and Refugee Children
Source: Int J Environ Res Public Health. 2021 Mar 28;18(7):3514. doi: 10.3390/ijerph18073514 (PMC8038070; doi:10.3390/ijerph18073514)
Supplement: Supplementary file 1 [file ijerph-18-03514-s001.pdf]

Supplementary materials

Table 1. Key Findings.

	Author	Year of Publication	Objectives	Key Findings
[112]	Meyer, S.R.; Meyer, E.; Bangirana, C.; Mangen, P.O.; Stark, L.	2019	To explore the potential influence of influx of refugees on adolescent well-being from South Sudan and those in Uganda	Influx impacts food security, hunger, education access and quality, psychosocial well-being, safety, violence, and injury.
[138]	Simeoni, S.; Frova, L.; De Curtis, M.	2019	To analyze the infant and neonatal mortality rates of Italian and foreign children and to evaluate if there is a disparity among geographical macro-areas	Data show disparity and reported neonatal (3.0 vs. 1.8/1000) and infant (4.5 vs 2.6/1000) mortality rates among foreign children compared to Italian children. The infant mortality among children born to women coming from Central and South Africa (8.2 /1000) is highest.
[125]	Williams, T.P.; Chopra, V.; Chikanya, S.R.	2018	To identify protection issues facing female adolescents in refugee camps	Vulnerabilities includes material deprivation, poverty, gender inequality, child abuse, transactional sex, and exploitation within and around the camps
[126]	Warria, A.	2018	To show challenges identified in assistance provision to child victims of transnational trafficking in South Africa	The challenges include ensuring safety, the diverse backgrounds and needs of trafficked children, matching lifestyle previously provided by pimps/traffickers, and premature return and reintegration of the children.
[183]	Metsaniitty, M.; Waltimo-Siren, J.; Ranta, H.; Fieuws, S.; Thevissen, P.	2018	To compare the age prediction performances of the Willems et al. model (WM) and the newly developed model collected from dental panoramic radiographs from Somali children living in Finland	Somali-specific age estimation model (SM) based on the WM resulted in a slight underestimation of age when the sex groups were analysed separately or combined, with mean error varying between 0.04 (standard deviation (SD) 1.01) and 0.05 (SD 1.04) years, mean absolute error between 0.77 and 0.80 years and root mean squared error between 1.01 and 1.04 years.
[50]	Nesheim, S.R.; Linley, L.; Gray, K.M.; Zhang, T.; Shi, J.; Lampe, M. A.; et al	2018	To understand the contribution of birthplace to the epidemiology of diagnosed HIV infection among US-born and foreign-born children	Of the 676 children <13 years born outside the United States but diagnosed with HIV infection in the United States, 443 (65.5%) were born in Africa.
[156]	Mor, Z.; Amit Aharon, A.; Sheffer, R.; Nehama, H.	2018	To compare development and growth achievements between children of Eritrean mothers (CE) to children of Israeli mothers (CI), and assess their compliance to routine follow-up and vaccination-timeliness	No statistically significant differences were found in birth anthropometric measurements. CE were more likely to fail in tests assessing fine-motor skill, linguistic and socio-emotional domains than CI, while no statistical difference was found in gross-motor achievements. 74.9% of the CE and 74.1% of the CI completed the vaccination schedule at follow-up.

[208]	Meyer-Weitz, A.; Oppong Asante, K.; Lukobeka, B.J.	2018	To explore refugee caregivers' perceptions of their children's access to quality health service delivery to their young children in Durban, South Africa	95% of caregivers were not satisfied with healthcare services delivery to their children due to the long waiting hours and the negative attitudes and discriminatory behaviours of healthcare workers, particularly in public healthcare facilities.
[195]	Chiappini, E.; Zaffaroni, M.; Bianconi, M.; Veneruso, G.; Grasso, N.; Garazzino, S.; et al	2018	To evaluate the prevalence of infectious diseases and immunisation status of children adopted from Africa	50.1% had at least one infectious disease. The most common parasitic infections were Giardia lamblia, Toxocara canis, and skin infections, notably Tinea capitis/corporis and Molluscum contagiosum.
[88]	Beukeboom, C.; Arya, N.	2018	To examine the variation among ethnic populations in prevalence of anemia and vitamin D and B12 deficiencies among refugee children	15.7% were anemic (25% < 5 years, 8.7% 5–11 years, and 18.3% 12–16 years old), with Somali children having the lowest hemoglobin levels compared to those from Iraq, Afghanistan, and Myanmar. 53.5% were vitamin D deficient seen most commonly in Iraqis and Afghans. 11.2% had vitamin B12 levels < 150 pmol/L.
[115]	Stevens, G.W.; Thijs, J.	2018	To investigate the associations between perceived group discrimination and several indicators of psychological well-being among Moroccan-Dutch adolescents	Higher perceived group discrimination was associated with higher parent- and adolescent-reported internalizing (e.g., fear, worries) and externalizing problems (e.g., anger, aggression) but not with lower personal self-esteem.
[43]	Grigoryan, K.V.; Tollefson, M.M.; Olson, M.A.; Newman, C.C.	2018	To evaluate the demographic characteristics, treatments, and resolution among pediatric patients with T. violaceum and T. soundance infections	81 children were identified as positive for T. violaceum and T. soudanense. Race/ethnicity of 67 patients (82.7%) was African. Of 28 patients not lost to follow-up, median time to clinical cure was 2.3 months.
[33]	Ndombo, P.K.; Ndze, V.N.; Mbarga, F.D.; Anderson, R.; Acho, A.; Ebua Chia, J.; et al	2018	To characterise measles virus among Central African Republic (CAR) refugees during the 2014 measles epidemic in Cameroon	Strains characterised were all genotype B3. Strains from genotype B3 found in this outbreak were more similar to those circulating in northern Cameroon in 2010–2011 than to MeV strains circulating in the CAR in 2011.
[80]	Mulugeta, W.; Glick, M.; Min, J.; Xue, H.; Noe, M. F.; Wang, Y.	2018	To examine longitudinal changes in obesity and overweight/obesity rates among resettled refugees and identify high risk subgroups	Obesity and overweight/obesity rates increased among refugees, but significant variations existed. African origin, baseline weight, and longer duration of stay in the USA were risk factors.
[102]	Aakre, I.; Henjum, S.; Folven Gjengedal, E.L.; Risa Haugstad, C.; Vollset, M.; Moubarak, K.; et al	2018	To explore concentrations of selected elements in distributed drinking water from households in the Saharawi refugee camps	Most elements in drinking water had higher concentration in zone 2 compared to zone 1 exceeded the WHO guidelines. Among both women and children, urinary concentration of vanadium, arsenic, selenium, lead, iodine, and uranium

				exceeded reference values, and most of the elements were significantly higher in zone 2 compared to zone 1. Among the children, 12% were underweight, 33% stunted, and 4% wasted.
[199]	Bawa, S.; Afolabi, M.; Abdelrahim, K.; Abba, G.; Ningi, A.; Tafida, S.Y.	2018	To describe the engagement process for a transboundary nomadic population and the interventions provided to improve population immunity among them while traversing through Nigeria	2015 children aged under 5 years were vaccinated with oral polio vaccines (OPV), of which 264 (13.1%) were zero-dose during the February 2016 polio supplemental immuninization activity (SIA) while, in the March immunization plus days (IPDs), 1864 were immunized of which 211 (11.0%) were zero-dose.
[114]	Zwi, K.; Woodland, L.; Williams, K.; Palasanthiran, P.; Rungan, S.; Jaffe, A.; et al	2018	To investigate protective factors for social-emotional well-being in refugee children in Australia	Social-emotional well-being mostly improves over time in refugee children. Children with four or more protective factors are at low risk of poor social-emotional well-being.
[62]	Belhassen-Garcia, M.; Pardo-Lledias, J.; Perez Del Villar, L.; Muro, A.; Velasco-Tirado, V.; Muñoz Bellido, J.L.;	2017	To describe the importance of relative eosinophilia in a cohort of immigrant children (<18 years) from sub-Saharan Africa, North Africa, and Latin America	Most children with parasitic infections with eosinophilia were from sub-Saharan Africa (especially West Africa) (99, 56.3%), followed by North Africa (54, 30.7%) and Latin America (25, 13.0%). The findings suggest that relative eosinophilia is frequently associated with helminthic infection in immigrant children from tropical and subtropical areas.
[162]	Esler, A.N.; Hall-Lande, J.; Hewitt, A.	2017	To examine differences across racial/ethnic groups in Autism Spectrum Disorder (ASD) symptoms, cognitive and adaptive skills, and related behaviors in children from the Somali diaspora	Somali children were more likely to have ASD with intellectual disability than children from all other racial/ ethnic groups.
[111]	Meyer, S.R.; Steinhaus, M.; Bangirana, C.; Onyango-Mangen, P.; Stark, L.	2017	To explore the relationship between caregiver depression and adolescent mental health in two refugee settlements in Uganda	Caregiver depression, gender, and exposure to violence were all associated with higher symptoms of adolescent depression in both sites and the full sample.
[70]	Woldeghebriel, M.; Hromi-Fiedler, A.; Lartey, A.; Gallego-Perez, D.; Sandow, A.; et al	2017	To explore the association between the amount of time living in Ghana and exclusive breastfeeding (EBF) practices among Liberian refugees and Ghanaians in surrounding villages	Increased time living in Buduburam improved the chances of EBF success among Liberians, perhaps as a result of unique EBF education/support opportunities offered in the settlement to Liberian refugees that were not readily available to Ghanaians.
[73]	Montero, M.D.P.; Mora-Urda, A.I.; Anzid, K.; Cherkaoui, M.; Marrodan, M.D.	2017	To compare the diet of adolescents of Moroccan origin living in Spain with that of adolescents living in Morocco	Teenagers from Morocco living in Madrid consumed more calories, proteins, saturated fats, and simple sugars (p<0.001) than those living in Morocco.

[204]	Aakre, I.; Strand, T. A.; Moubarek, K.; Barikmo, I.; Henjum, S.	2017	To explore whether young children's developmental status is associated with thyroid dysfunction in an area of chronic excessive iodine exposure	The high iodine intake may have caused the thyroid dysfunction and hence the delayed developmental status.
[150]	Ortiz-Echevarria, L.; Greeley, M.; Bawoke, T.; Zimmerman, L.; Robinson, C.; Schlecht, J.	2017	To explore the factors and risks that impact the health of very young adolescents (VYAs), those 10–14 years of age, in Kobe Refugee camp	The risk factors include newfound access to education and security, combined with gender divisions and parental communication around early sexual and reproductive health (SRH) and puberty that remained intact from traditional Somali culture.
[163]	Fox, F.; Aabe, N.; Turner, K.; Redwood, S.; Rai, D.	2017	To assess what families affected by autism need, and how health, education, and social care services can support Somali migrants	Two themes are reported; 'Perceptions of Autism' and 'Navigating the System'. For Somali families in this study, cultural attitudes towards mental illness, challenging behaviour and disability, combined with the lack of vocabulary to describe and explain autism made the understanding and acceptance of their child's autism particularly difficult.
[108]	Aviad-Wilchek, Y.; Ben-David, I.L.	2017	To explore the relationship between parental immigration-related trauma and second-generation adolescent substance abuse	Readiness levels among Ethiopian adolescents to use psychoactive substances are relatively low, and that parental trauma only affects the readiness to consume alcohol. The levels of readiness to consume drugs were partially related to parental trauma.
[210]	Zwi, K.; Morton, N.; Woodland, L.; Mallitt, K. A.; Palasanthiran, P.	2017	To determine the prevalence of health conditions in newly arrived refugee children, from Africa, Southeast Asia, Eastern Mediterranean, Europe/Western Pacific, and access to timely health screening in regional Australia	97% of arrivals have access to primary care screening and 72% have completed all recommended screening tests.
[189]	Bennet, R.; Eriksson, M.	2017	To describe the results of tuberculosis (TB) screening of asylum-seeking, unaccompanied minors with a stated age <18 years arriving in Sweden in 2015	26–32% of latent TB infection and 3.4–3.5% of TB among those from Eritrea, Ethiopia, or Somalia.
[119]	Abdullahi, I.; Kruger, E.; Tennant, M.	2017	To examine the service accessibility of Somali Australians suffering Autism Spectrum Disorder (ASD)	A quarter of children were within 2,000 m of a speech pathology service access point and nearly a third (31%) within 2,000 m of a psychologist.
[96]	Tondeur, M.C.; Salse, U.N.; Wilkinson, C.; Spiegel, P.; Seal, A.J.	2016	To assess the acceptability and adherence to daily doses of lipid-based nutrient supplement (LNS) amongst children and micronutrient powder (MNP) amongst children and pregnant and lactating women	98.4% of LNS children, 90.4% of MNP women, and 75.5% of MNP-children ($P<0.05$) liked the product.

[39]	Mashiah, J.; Kutz, A.; Ami, R.B.; Savion, M.; Goldberg, I.; Or ,T.G.; et al	2016	To determine epidemiologic trends for Tinea capitis (TC) among paediatric populations at the Tel Aviv Medical Center	An increase in TC rates was noted over the study period, with higher rates in boys than girls. Trichophyton violaceum (TV), Trichophyton soudanense (TS) and Microsporum audouinii (MA) were the prominent causative organisms
[71]	Aakre, I.; Strand, T.A.; Bjoro, T.; Norheim, I.; Barikmo, I.; Ares, S.; Alcorta, M.D.; Henjum, S.	2016	To describe iodine status among breastfed infants with high iodine exposure in the Saharawi refugee camps Algeria, and thyroid function and iodine status three years later	At baseline and follow-up, 88% and 72% had excessive iodine intakes (UIC ě 300 μg/L), respectively. At follow-up, 24% had a thyroid hormone disturbance and/or elevated serum Tg, including 9% with subclinical hypothyroidism (SCH), 4% with elevated fT3 and 14% with elevated Tg. Children with SCH had poorer linear growth and were more likely to be underweight than the children without SCH.
[89]	Khayri, H.O.; Muneer, S.E.; Ahmed, S.B.; Osman, M.A.; Babiker, E.E.	2016	To assess the nutritional status of primary school Sudanese pupils and explore its correlates	31% of respondents suffer from underweight and 6.75% from overweight. Participants' average daily intake of calories and fiber was significantly lower than that of the dietary requirement intakes (DRI) (1, 397.89 kcal versus 2,000 kcal, p=<0.01), some vitamins and iron was significantly higher than that of the DRI (p=<0.01).
[66]	Wandel, M.; Terragni, L.; Nguyen, C.; Lyngstad, J.; Amundsen, M.; de Paoli, M.	2016	To explore infant feeding practices among Somali-born mothers in Norway, and the ways in which they navigate among different information sources	The mothers had positive attitudes to breastfeeding but were unfamiliar with the concept of exclusive breastfeeding. Early introduction of water and infant formula was a common practice that interfered with exclusive breastfeeding.
[124]	Iyakaremye, I.; Mukagatare, C.	2016	To explore the situation of sexual abuse of Congolese adolescent girls in Kigeme camp and to suggest remedial strategies	The findings show that rape, unwanted physical touching, sexual exploitation, commercial sex, early marriage, and girl trafficking are the main forms of sexual abuse.
[165]	Mude, W.; Mwanri, L.	2016	To explore parents' perspectives of barriers to participation in physical activity among South Sudanese children in South Australia	For some disadvantaged migrant communities, especially those of refugee backgrounds. Experiences of low income and being recent arrivals with no or little English may also increase and compound vulnerability to physical inactivity.
[143]	Racape, J.; Schoenborn, C.; Sow, M.; Alexander, S.; De Spiegelaere, M.	2016	To analyse national Belgian data to determine 1) whether socioeconomic status (SES) modifies the association between maternal nationality and perinatal outcomes (low birth weight (LBW) and perinatal mortality);	Compared to Belgians, an increased risk of perinatal mortality was observed in all migrant groups ($p < 0.0001$), despite lower rates of LBW in some nationalities. Sub-Saharan African mothers had a significantly higher risk of LBW

				compared to Belgians. Acquisition of the Belgian nationality significantly decreased the mortality odds for all the nationalities.
[103]	Dawson-Hahn, E.E.; Pak-Gorstein, S.; Hoopes, A.J.; Matheson, J.	2016	To describe the prevalence of wasting, stunting, overweight, and obesity among refugee children (from Iraq, Somalia, and Burma) ages 0–10 years prior to resettlement in Washington State (WA)	Overall, nearly one-half of all refugee children had at least one form of malnutrition (44.9%). Refugee children ages 0–10 years were affected by wasting (17.3%), stunting (20.1%), overweight (7.6%), and obesity (5.9%). Wasting was significantly higher among young Somali (23.2%, $p<0.001$) vs. low-income WA children (1.9%).
[67]	Grewal, N.K.; Andersen, L.F.; Sellen, D.; Mosdol, A.; Torheim, L.E.	2016	To examine breastfeeding and complementary feeding practices during the first 6 months of life among Norwegian infants of Somali and Iraqi family origin	Only 7% of Norwegian-Somali and 10% of Norwegian-Iraqi infants were exclusively breastfed at 4 months of age. By 1 month of age, water had been introduced to 30% of Norwegian-Somali and 26% of Norwegian-Iraqi infants, and infant formula to 44 and 34%, respectively.
[76]	Grewal, N.K.; Andersen, L.F.; Kolve, C.S.; Kverndalen, I.; Torheim, L.E.	2016	To describe food and nutrient intake among 12-month-old Norwegian-Somali and Norwegian-Iraqi infants, with a focus on iron and vitamin D intake	40% of the Norwegian-Somali infants and 47% of the Norwegian-Iraqi infants were breastfed at 12 months of age. Most of the infants consumed iron-enriched products (81%) and received vitamin D supplements (84%).
[170]	Gorrab, A.A.; Fournier, A.; Bouaziz, A.A.; Spigelblatt, L.; Scuccimarri, R.; Mrabet, A.; Dahdah, N.	2016	To compare the incidence of Kawasaki disease in Maghrebi children living in Quebec to the countries of origin, Morocco, Algeria, and Tunisia	The annualized incidence rate in Quebec (18.49/year/100000 children under 5 years of age) was 4 to 12 times higher than in Tunisia, Morocco, and Algeria (0.95, 4.52, and 3.15, respectively).
[51]	Neubert, J.; Michalsky, N.; Laws, H.J.; Borkhardt, A.; Jensen, B.; Lubke, N.	2016	To determine the HIV-1 subtype distribution and the prevalence of drug resistant mutations (DRMs) in therapy-naïve HIV-1-infected children who received routine care at the University Hospital Düsseldorf, Germany	More than half of the HIV-1 children (66%) had one or both parents of African origin.
[137]	Vang, Z.M.	2016	To compare neonatal and postneonatal mortality between the Canadian-born population and 12 immigrant subgroups were compared using 1990–2005 linked birth-infant death records	The adjusted risk of neonatal mortality was higher for sub-Saharan African (hazard ratio [HR] = 1.32; 95 % confidence interval [CI] = 1.05, 1.66), Haitian (HR = 2.29, 95 % CI = 1.90, 2.76), non-Spanish Caribbean (HR = 1.38; 95 % CI = 1.01, 1.89), and Pakistani (HR = 1.87; 95 % CI = 1.31, 2.68) migrants relative to Canadian-born women. Improvements in prenatal care and access to postpartum care

				may reduce disparities in infant mortality.
[72]	Aakre, I.; Lilleengen, A.M.; Lerseth Aarsand, M.; Strand, T.A.; Barikmo, I.; Henjum, S.	2016	To describe breastfeeding and general feeding practices and the nutrition status among children from birth to 6 months of age in the Saharawi refugee camps located in Algeria	13.8, 8.2, and 16.5% of the infants were stunted, wasted, and underweight, respectively. Approximately 65% initiated breastfeeding within 1 h after birth and 11.7 and 21.6% were exclusively or predominantly breastfed less than 6 months.
[48]	Feiterna-Sperling, C.; Konigs, C.; Notheis, G.; Buchholz, B.; Kruger, R.; Weizsacker, K.; Eberle, J.; Hanhoff, N.; Gartner, B.; Heider, H.; Kruger, D. H.; Hofmann, J.	2016	To determine the seroprevalence of HHV-8 among vertically HIV-1-infected pediatric patients living in Germany and to evaluate its association with HIV disease, age, gender, ethnicity, and other demographic factors.	The overall HHV-8 seroprevalence was 24.6 % without significant differences related to sex, age, or ethnicity. HIV-infected children with African or mixed (African/Caucasian) ethnicity had a higher seroprevalence (33/111; 29.7 %) compared to the other patients, with Caucasian, Asian, or Hispanic ethnicity (18/96; 18.8 %), but this did not reach statistical significance ($p = 0.076$). In multivariate analysis, a child's birth outside Germany was the only significant risk factor for HHV-8 seropositivity (OR 3.98; 95 % CI [1.27–12.42])
[192]	Nejat, S.; Bennet, R.	2016	To evaluate positive tuberculin skin tests (TSTs) and interferon-gamma release assays (IGRAs) on paediatric migrants in relation to the TB incidence in the corresponding foreign-born populations in Stockholm	IGRA positivity ranged from 64% in migrants from Somalia to 20% in those from the former Soviet Union and eastern Europe. IGRAs showed higher specificity than TST in identifying tuberculosis.
[181]	Svensson, I.; Gustafsson, J.; Uleskog, E.; Mathisson, C.; Mollai, N.; Kahlmeter, A.; Matsson, L.	2016	To obtain baseline information about oral health, oral health behaviours, and knowledge about prevention of oral diseases in newly arrived Somali children	78–82% of the children in the three groups had never visited a dental clinic in Somalia. Toothache was reported by 18–28% of the children. About half the children had poor oral hygiene while 25–33% reported daily intake of sugary snacks. Malocclusion affected 40% of the 7–11-year-olds and 50% of the 12–17-year-olds.
[113]	Jensen, T.K.; Fjermestad, K.W.; Granly, L.; Wilhelmsen, N.H.	2015	To examine life experiences and mental health among unaccompanied asylum seekers (from Asia and Africa) aged 16 years and younger, who were newly arrived, and still in an asylum-seeking process and thus in temporary residency	Participants from Asian countries had experienced an average of 1.1 more stressful life events than participants from African countries.
[75]	Toselli, S.; Zaccagni, L.; Celenza, F.; Albertini, A.; Gualdi-Russo, E.	2015	To assess the differences in adiposity, lifestyle habits, and parents' features in preschool children according to their parental migration status and to evaluate the influence of	African males had a significantly lower prevalence of normal weight than Italian males (50.0 vs. 70.9%), as well as the highest frequency of overweight and obesity (25.0 and

			behavioral/lifestyle factors on the children's BMI	19.4%, respectively). Daily breakfast (''every day'') was most common in Italians and Other Europeans, with significant differences between Italian and African females (83.3 vs. 62.9%, respectively).
[168]	Bakken, K.S.; Skjeldal, O.H.; Stray-Pedersen, B.	2015	The aim of this study was to examine differences in distribution of neonatal jaundice by migration indicators	3.7%% African infants had neonatal jaundice compared to 6.4% Norwegian infants ($p<0.007$). African infants were more often transferred to a neonatal intensive care unit (NICU) compared to Norwegian infants (31.8 vs. 11.6%, $p=0.013$).
[110]	Wilchek-Aviad, Y.	2015	To look at the connection between meaning in life and suicide among youth born to immigrants from a developing country, in Ethiopia—compared to native-born Israeli youth	A significant negative correlation between meaning in life and suicidal tendencies was found for Ethiopian youths ($r=-0.61$, $p<0.001$). Higher suicidal tendencies, anxiety, and depression were found among immigrants compared to native-born Israelis, with immigrant boys displaying more anxiety than girls.
[179]	Amin, M.; Perez, A.; Nyachhyon, P.	2015	To assess parental awareness of their child's dental status and the relationship between parental awareness and children's dental attendance	52% of the children never had a dental visit. Dental status of 44% of children was rated as good by parents; among them, 56% had dental decay. Parental assessments did not coincide with the clinical assessments of 62 % of children.
[122]	Inguglia, C.; Musso, P.	2015	a) What are the acculturation profiles of immigrant adolescents? b) What are the acculturation expectation profiles of autochthonous adolescents? c) How are these intercultural profiles related to adaptation of both immigrant and autochthonous adolescents?	Immigrant adolescents were divided in two acculturation profiles, ethnic and integrated-national, with adolescents belonging to the latter showing higher self-esteem, life satisfaction, and sociocultural competence than the former.
[95]	Renzaho, A.M.; Halliday, J.A.; Mellor, D.; Green, J.	2015	To develop a culturally-competent obesity prevention program for sub-Saharan African (SSA) families with children aged 12–17 years population in Australia	The 'Healthy Migrant Families Initiative (HMFI): Challenges and Choices' program was developed and divided into two modules: 1) 'Healthy lifestyles in a new culture' and 2) 'Healthy families in a new culture'
[188]	Veenstra-van Schie, M.T.; Coenen, K.; Koopman, H.M.; Versteegh, F.G.	2015	To explore the differences in the perceived HRQoL between children with asthma from Moroccan and Dutch descent and their parents	Moroccan children differed significantly on the DUX-25 overall score ($p = 0.002$) and the subscales physical ($p = 0.006$) (Table 1, lower part), emotional ($p=0.003$), and home functioning ($p = 0.006$) also indicating a better health related quality of life compared to Dutch children.

[104]	Ercolini, D.; Francavilla, R.; Vannini, L.; De Filippis, F.; Capriati, T.; Di Cagno, R.; Iacono, G.; De Angelis, M.; Gobbetti, M.	2015	To investigate the salivary microbiota of the Saharawi population how they are influenced by the intake of specific nutrients	A remarkable change in the metabolic potential of the microbiota occured following the diet change, with increased potential for amino acid, vitamin and co-factor metabolism. High concentrations of acetone and 2-butanone were noted during treatment with the Italian-style gluten-free diet, which suggested metabolic dysfunction in the Saharawi celiac children.
[167]	Thul, C.M.; Eisenberg, M.E.; Larson, N.I.; Neumark-Sztainer, D.	2015	To assess the levels and types of physical activity among a population-based sample of Somali and other non-Hispanic black and white adolescents living in Minnesota	Somali girls had lower mean moderate-to-vigorous-physical-activity (MVPA) hours than their peers; however, no differences were found for Somali boys. Involvement in most activities was similar for Somali and other groups, but some differences were observed. For example, Somali youth were more likely to play soccer than their same-sex other black peers (boys: 52.4 vs. 20.4%; girls: 34.6 vs. 14.6%; p<. 05).
[47]	Cohen, S.; van Bilsen, W. P.; Smit, C.; Fraaij, P. L.; Warris, A.; Kuijpers, T. W.; Geelen, S. P.; Wolfs, T. F.; Scherpbier, H. J.; van Rossum, A. M.; Pajkrt, D.	2015	To investigate potential differences in clinical, immunological, and virologic outcome in Dutch HIV-infected children born in the Netherlands (NL) versus born in sub-Saharan Africa (SSA) in a national cohort analysis	Most immigrant HIV-infected children living in NL were born in SSA (47%). Children born in SSA were diagnosed and initiated cART at an older age (3.7 years, IQR:0.9–9.0) ($p < 0.001$), than children born in NL ((1.2 years, inter quartile range (IQR):0.3–3.7). Despite initial differences in CD4+ T-cell counts and HIV viral load, there was no difference in immunological reconstitution during and after the first 5 years of cART between the 2 groups ($p < 0.05$).
[175]	Quach, A.; Laemmle-Ruff, I.L.; Polizzi, T.; Paxton, G.A.	2015	To report on oral health in refugee-background children in Australia, and to assess their follow-up at dental services	Compared to Australian-born children of African background, African-born children were more likely to be referred for further dental care (adjusted PR 1.33, 95% CI [1.02 – 1.73]) although there was no statistically significant difference in caries prevalence.
[214]	Corriveau-Bourque, C.; Bruce, A.A.	2015	To measure the prevalence of sickle cell disease and thalassemia in the Northern Alberta pediatric population and to determine the clinical outcomes.	25% of patients born abroad originated from Africa. African participants had sickle cell diagnoses of HbSS, HbS/B+thalassemia, or HbSC
[107]	Völkl-Kernstock, S.; Karnik, N.; Mitterer-Asadi, M.; Granditsch, E.; Steiner, H.; Friedrich, M.H.; Huemer, J.	2014	To investigate African unaccompanied refugee minors (URMs) living in Austria for posttraumatic stress disorder (PTSD) prevalence and related symptoms and coping strategies	The study revealed lower PTSD rates than measured among URMs in previous studies. Girls were more likely to develop PTSD. PTSD was significantly correlated with single war-related traumatic events. The depression score for the sample was above the clinical cut-off value.

[153]	Macipe-Costa, R.M.; García-Sanchez, N.; Gimeno-Feliu, L.A.; Navarra-Vicente, B.; Jiménez-Hereza, J.M.; Moneo-Hernández, I.; et al	2014	To study the frequency, characteristics, and complications of non-therapeutic male circumcision on immigrant children from Africa in Spain	The results obtained in this study confirm the significance of non-therapeutic male circumcision among the African immigrant population in Spain. Half (49.1%) of the families who had the circumcision performed in Europe did so at home. 26 of the children in the study were circumcised at home, with 22 thereof from Gambia and the rest from Ghana, Mali, Guinea-Conakry, and Morocco.
[69]	Tyler, L.; Kirby, R.; Rogers, C.	2014	To highlight and compare immigrant Sudanese women's infant feeding choices and patterns before and after moving to a regional city in Queensland, Australia	The study indicated a trend for immigrant women's breastfeeding duration to decline when they moved to another country. This research suggests that the reasons for this decline are complex. The authors conclude that a lack of social support, language difficulties, and wanting to fit in with particular Western practices are contributing factors.
[209]	Soriano, E.; Ruiz, D.; Higginbottom, G.	2014	To analyze how the health promotion concept is integrated into the ethos of the Moroccan population through the positions set forth with respect to adherence to the Healthy Child Program	Moroccan mothers maintained the belief that it is pointless to go to health professionals; the women perceived themselves as responsible for child care and health, and for that reason assumed some options of the host society whenever they considered it to be good for the child and did not clash with ideas, values, and uses of their culture.
[65]	Shah, A.Y.; Suchdev, P.S.; Mitchell, T.; Shetty, S.; Warner, C.; Oladele, A.; Reines, S.	2014	To determine the nutritional status among refugee children entering one of the largest resettlement countries in the United States and identify differences between incoming populations	African refugees had a higher prevalence of stool parasites (34.6%) compared to Bhutanese (20.9%) and Burmese (24.4%) refugees (p = 0.06). Underweight prevalence of the African refugees (10.0%) was much lower than both Bhutan and Burmese refugees (p = 0.02). The stunting prevalence among African refugees <18 years (9.1%) was significantly lower than among refugees from Bhutan (19.0%) or Burma (22.0%) (p = 0.03).
[131]	Stevens, G.W.; Veen, V.C.; Vollebergh, W.A.	2014	To compare bidimensional psychological acculturation orientation of Moroccan immigrant boys in pretrial detention with those of Moroccan boys in the general population	Parents with sons in pretrial detention were more likely to be divorced than parents from the general population (14 vs. 4%, respectively). Boys in pretrial detention had dropped out of school much more often than boys from the general population (22 vs. 4%, respectively). Integrated boys were found to be almost five times as likely to belong to the pretrial detention group than separated boys

				(OR 4.7, CI [2.0, 11.2], p < .00), whereas no significant differences occurred between ambivalently acculturated and separated boys (OR 1.9, CI [0.5, 6.9], p .34).
[141]	Juarez, S.P.; Revuelta-Eugercios, B.A.	2014	To compare the main indicators related to birth weight and gestational age (LBW, macrosomia, preterm and post-term) for immigrants and Spaniards	Sub-Saharan immigrants to Spain showed higher risks in all perinatal outcomes studied (LBW, macrosomia, preterm and post-term births)
[187]	Shani, M.; Band, Y.; Kidon, M.I.; Segel, M.J.; Rosenberg, R.; Nakar, S.; Vinker, S.	2013	To compare the prevalence of asthma among Israeli-born children of Ethiopian origin to that in non-Ethiopian children	More Ethiopian children came from families with a low socioeconomic status (23.9 vs. 17%,), and with significantly lower parental smoking (5.1 vs. 40.1%). The prevalence of asthma was 92/1217 (7.5%) among children of Ethiopian origin, compared to 122/1217 (10.0%) among the control group. When adjusted for tobacco exposure, the OR for risk of asthma in the Ethiopian children was 0.80 (95% CI: 0.59e1.09,).
[172]	Davidovich, E.; Kooby, E.; Shapira, J.; Ram, D.	2013	To assess whether the prevalence of missing primary canines and dental defects in offspring of emigrants from Ethiopia is greater than in offspring of native Israeli parents of similar socioeconomic class	Canines were present in more Israeli than Ethiopian younger children, 87.5 vs. 42.3%, p=0.0001; and in more Israeli than Ethiopian older children, 92.6 vs. 40.4%, p=0.0001. More dental defects were detected in Ethiopian than in Israeli younger children, 32 vs. 3.9%, p=0.0001; and in more Ethiopian than Israeli older children, 31.2 vs. 5.8%, p=0.0001.
[201]	Hussen, H.I.; Moradi, T.; Persson, M.	2015	To investigate the effect of the mother's duration of stay in Sweden on the risk of type 1 diabetes mellitus (T1DM) in the offspring	Offspring of mothers living in Sweden for up to 5 years had a 22% lower risk of T1DM (IRR 0.78, 95% CI 0.63–0.96) compared with offspring of mothers living in Sweden for at least 11 years. Offspring of mothers from eastern Africa had the same risk pattern with increasing risks with longer duration of stay. Children born to eastern African mothers living in Sweden for more than 11 years had a doubled risk of T1DM compared with native Swedes (IRR 2.27, 95% CI 1.25–4.10).
[178]	Davidovich, E.; Kooby, E.; Shapira, J.; Ram, D.	2013	To compare oral hygiene habits in preschool children from low socioeconomic neighborhoods offspring of immigrants from Ethiopia to offspring of native Israelis	Of children aged 49-82 months, 15% offspring of Ethiopian and 25% of native Israelis were reported to have visited a dentist; and 45% and 65%, respectively, to brush their teeth at least once daily. More than 90% of children of both populations were reported to have toothbrushes. Of children aged 18-48 months, 28% of Ethiopian and 65% of native Israelis

				were reported to brush their teeth at least once daily.
[123]	Sanchez-Cao, E.; Kramer, T.; Hodes, M.	2013	This study aims to describe the level of psychological distress among a group of unaccompanied asylum-seeking children (UASC) and the pattern of mental health service (MHS) contact	Unaccompanied asylum-seeking children were mainly Black African, 39/71 (54.9%). 15.4% of African UASC had MHS contact, compared to 84.6% of African UASC did not have contact with MHS.
[200]	Cadario, F.; Bruno, G.; Cerutti, F.; Savastio, S.; Tumini, S.	2013	To assess the burden and clinical features of type 1 diabetes in migrants with respect to Italian children	All African migrants had T1D onset in Italy.
[92]	Polonsky, J.A.; Ronsse, A.; Ciglenecki, I.; Rull, M.; Porten, K.	2013	To present the survey estimates of death rates and malnutrition prevalence, and the age and sex breakdown of the suspected measles cases, and discuss the implications of the findings	The under-5 death rate was 1.8 per 10,000 person-days. More than two-thirds of all deaths were reported were associated with diarrhoea (25%), cough or other breathing difficulties (24%), or with fever (19%). Measles accounted for 17% of all deaths; this was due to a measles outbreak. Trends of decreasing death rates and malnutrition prevalence with length of stay in Bulo Bacte were observed.
[169]	Montanaro, M.; Colombatti, R.; Pugliese, M.; Migliozzi, C.; Zani, F.; Guerzoni, M. E.; et al	2013	To describe the intellectual function of first-generation African immigrants with sickle cell disease (SCD) and the influence of sociodemographic factors on its characteristics	Immigrant bilingual children with SCD seem to display a rate of cognitive impairment similar to their monolingual counterparts but a more pronounced and precocious onset of language difficulties.
[164]	Wijtzes, A. I.; Jansen, W.; Jaddoe, V. W. V.; Moll, H. A.; Tiemeier, H.; Verhulst, F. C.; et al	2013	To evaluate the association between ethnic background and children's TV viewing time at multiple time points and its trajectory	After adjustment for maternal educational level and household income, Moroccan children (aOR, 1.68; 95% CI, 1.03–2.76) were significantly more likely to watch television for 2 or more hours/day compared with native Dutch children.
[121]	Ndengeyingoma, A.; de Montigny, F.; Miron, J.M.	2013	To examine what contributes to the development of personal identity among refugee adolescents of African origin who have immigrated to Québec	The elements contributing to personal identity are divided into three categories: (1) personal characteristics (capacity for self-criticism, religious beliefs, complex migratory paths), (2) interpersonal relationships (relationships with peers, family dynamics), and (3) environmental characteristics.
[155]	Vang, Z. M.; Elo, I.T.	2013	To explore the association between neighborhood racial diversity and black immigrant perinatal health	Minority diversity had a protective effect on black infant health. Women living in low and moderately diverse tracts as well as those in majority-minority neighborhoods had heavier babies (β=26.5, 29.8 and 61.2, respectively, $p<0.001$) on average than women in the least diverse tracts.

[84]	Kaufman-Shriqui, V.; Fraser, D.; Friger, M.; Bilenko, N.; Vardi, H.; Abu-Saad, K.; et al	2013	To examine the relationship between acculturation and obesity among low socioeconomic status (LSES) children	Smoking increased children's risk of obesity.
[193]	Mor, Z.; Cedar, N.; Pinsker, G.; Bibi, H.; Grotto, I.	2013	The study describes the characteristics of childhood TB in Israel and examines treatment outcomes	Of all childhood TB cases, 236 (56.7%) were born in countries with high prevalence of TB; 214 (51.5% of all children) were Israeli citizens who were Ethiopian-born. In 2010, the incidence of childhood TB if at least one of their parents was Ethiopian-born was 7.5 cases per 100,000 children.
[154]	Pruitt, C.N.; Reese, C.S.; Grossardt, B.R.; Shire, A.M.; Creedon, D.J.	2013	To determine whether Somali girls living in a US community are following the recommendations for HPV vaccination	Somali girls to be generally accepting of initiating the HPV vaccine series but less likely to complete the series as compared with white non-Hispanic girls of the same age.
[186]	Patel, A.R.; Zietlow, J.; Jacobson, R.M.; Poland, G.A.; Juhn, Y.J.	2013	To assess the prevalence of asthma and other atopic conditions in Somali immigrants and to determine the humoral immune response to the measles, mumps, and rubella (MMR) vaccine viruses in Somali immigrants with asthma	Study results may not support the hygiene hypothesis. Of the subjects, 10 (16%) had asthma and 22 (35%) had other atopic conditions. There was no difference in the rubella ($p=0.150$) and measles ($p=0.715$) virus-specific IgG levels between the subjects with and without asthma. Mumps virus-specific IgG antibody levels were lower in those with asthma than in those without asthma (mean±SE 2.08±0.28 vs. 3.06±0.14, $p=0.005$).
[109]	Walsh, S.D.; Edelstein, A.; Vota, D.	2012	To explore the relationship between ethnic identity (Israeli and Ethiopian) and parental support with suicide ideation and alcohol use	There is a significant relationship between suicide ideation and parental support. Positive Ethiopian heritage identity is associated with lower levels of suicidal ideation and alcohol use. Positive ethnic identity is an important protective factor against suicidal and risk behaviors.
[77]	Mescher-Benbeneck, M.; Garwick, A.W.	2012	To explore the dietary factors that contribute to bone health among first generation Somali girls and to determine the social and cultural contexts that shape these health behaviors	Cultural tradition, developmental stage, acculturation, and environment were major factors contributing to the intake of calcium and vitamin D-rich foods.
[116]	Beiser, M.; Taa, B.; Fenta-Wube, H.; Baheretibeb, Y.; Pain, C.; Araya, M.A.	2012	To examine the specificity of these putative mental health risks to the immigration experience. The level and predictors of emotional problems among preadolescent Ethiopians living in immigrant families in Toronto, Canada	Youth reported higher levels of emotional problems ($M=10.09$, $SD=2.56$) than their parents ($M=9.46$, $SD=2.10$). Predictors differed for parents and youth ratings. Contrary to immigration stress theory, parental perceptions of prejudice did not predict emotional problems. Perceived discrimination was a significant predictor of self-rated emotional problems.

[182]	Amin, M.; Perez, A.	2012	To identify psychosocial barriers to providing and obtaining preventive dental care for preschool children among African recent immigrants	Early childhood caries (ECC) were associated with barriers to parental prevention knowledge and self-efficacy for early detection as well as barriers to access.
[203]	Oilinki, T.; Otonkoski, T.; Ilonen, J.; Knip, M.; Miettinen, P. J.	2012	To compare the prevalence and characteristics of diabetes between Somali and Finnish children in the City of Helsinki	Somali children have autoimmune diabetes. The prevalence of T1D is similar among Somali (40/10 000 (95% CI 19–73/10 000)) and Finnish children (37/10 000 (95% CI 33–41/10 000)). Both affected and unaffected Somali children have low concentrations of S25(OH)D.
[117]	Astell-Burt, T.; Maynard, M. J.; Lenguerrand, E.; Harding, S.	2012	To investigate the effect of racism, own-group ethnic density, diversity, and deprivation on adolescent trajectories in psychological well-being	Ghanians/Nigerians had significantly more experiences of racism than their white peers at ages 11-13 years ($p<0.01$) and 14-16 years ($p<0.001$). Other Africans had significantly more experiences of racism than their white peers at ages 11-13 years ($p<0.001$) and 14-16 years ($p<0.01$).
[87]	Sheikh, M.; Wang, S.; Pal, A.; MacIntyre, C.R.; Wood, N.; Gunesekera, H.	2011	To determine the prevalence of vitamin D deficiency in refugee children in Sydney and to investigate risk factors	62 of the African children had no deficiency, 83 had mild deficiency, 112 had moderate deficiency, and 3 had severe deficiency. Deficiency was commonest among East African (72%, 70/97) and least common among those from West Africa (39%, 14/36). The odds ratio of deficiency in East Africa to the rest of Africa was 3:36:1.
[100]	Ndemwa, P.; Klotz, C.L.; Mwaniki, D.; Sun, K.; Muniu, E.; Andango, P.; et al	2011	To evaluate the effect of the availability of home fortification with a micronutrient powder containing 2.5 mg of sodium iron ethylenediaminetetraacetate (NaFeEDTA) on iron status and hemoglobin in women and children in the Kakuma Refugee Camp in northwest Kenya	In children and in women of childbearing age, the availability of micronutrient powder was associated with a small improvement in iron status but no significant change in hemoglobin in this refugee camp setting.
[128]	Paalman, C.H.; van Domburgh, L.; Stevens, G.W. J.M.; Doreleijers, T.A.H.	2011	To investigate which risk factors are associated with (re-)offending of childhood offenders from an ethnic minority (Dutch-Moroccan migrant children in the Netherlands)	All groups (controls, one-time offenders, and re-offenders) showed substantial problems. Single parenthood (OR 6.0) and financial problems (OR 3.9) distinguished one-time offenders from controls. Reading problems (OR 3.8), having an older brother (OR 5.5), and a parent having Dutch friends (OR 4.3) distinguished re-offenders from one-time offenders. The control group reported high levels of emotional problems (33.3%).
[149]	Flynn, P.M.; Foster, E.M.; Brost, B.C.	2011	To investigate the effect of indicators of acculturation among Somali refugee women's birth outcomes	Factors selected to reflect acculturation did not yield statistically significant relationships with preterm and low birth weight infants in this study. However, the magnitude of observed increases in

				risk factors contributing to poor birth outcomes are of concern in Somali refugee women, and the value of early and regular prenatal care was confirmed.
[194]	Paxton, G.A.; Rice, J.; Davie, G.; Carapetis, J.R.; Skull, S.A.	2011	To provide data on immunisation status of recently arrived East African children and adolescents in Australia	97% of the African immigrant children had either an incomplete or uncertain immunisation status. Low numbers of participants had serological immunity against hepatitis B (33%), diphtheria (45%), and tetanus (61%), and a higher number of children had immunity to measles (90%), rebella (77%), and tetanus (61%). Four percent of written vaccination records were available.
[166]	Thul C.M.; LaVoi, N.M.	2011	To explore the experiences with and beliefs about physical activity of East African adolescent female participants and suggestions for promoting active living	Deductive and inductive content analysis revealed that East African girls desired to be physically active and perceived a wide range of physical activities to be culturally relevant and desirable, yet faced an array of personal, social, environmental, and cultural barriers to active living.
[78]	Dharod, J.M.; Croom, J.E.	2010	To estimate the prevalence of child hunger and its relationship to dietary intake and body mass index (BMI) among Somali refugee children	Underweight or BMI < 5th percentile was significantly higher in children who experienced hunger. In terms of dietary intake, compared to food secure or mild to moderate food insecure households, intake of green leafy vegetables was significantly lower while intake of eggs was higher among children experiencing hunger.
[37]	Cherian, S.; Burgner, D.P.; Cook, A.G.; Sanfilippo, F.M.; Forbes, D.A.	2010	To investigate infection, gastrointestinal symptoms, and systemic inflammation these parameters in refugee children with a high prevalence of Helicobacter pylori, helminth, and malaria infection	There was a high prevalence of asymptomatic H. pylori infection in recently resettled African refugee children. Gastrointestinal symptoms were not predictive of H. pylori nor of helminth infections. Serum cytokines, particularly IL-5, IL-10, and TNFa, were significantly elevated in children with malaria and helminth infections but not in those with H. pylori infection.
[205]	Henjum, S.; Barikmo, I.; Gjerlaug, A.K.; Mohamed-Lehabib, A.; Oshaug, A.; Strand, T.A.; Torheim, L.E.	2010	To assess the prevalence of enlarged thyroid volume (Tvol) in Saharawi refugee children, and their urinary iodine concentration (UIC), and to identify possible sources of excess iodine intake	Enlarged Tvol was found in 56% (Tvol-for-age) and 86% (Tvol-for-body-surface-area) of the children. The median (25th percentile–75th percentile, P25–P75) UIC was 565 (357–887) mg/L. The median (P25–P75) iodine concentration in household drinking water was 108 (77–297) mg/L. None of the children had UIC below 100 mg/L, 16% had UIC between 100 and 299 mg/L, and 84% had UIC above 300 mg/L.

[135]	Racape, J.; De Spiegelaere, M.; Alexander, S.; Dramaix, M.; Buekens, P.; Haelterman, E.	2010	To describe and measure inequalities in perinatal mortality and causes of perinatal deaths according to maternal nationality and socioeconomic status	The excess in perinatal mortality rates for women of sub-Saharan Africa reflected a high rate of preterm deliveries and low birth weight, as well as a low socioeconomic level.
[52]	Arnaez, J.; Roa, M.A.; Albert, L.; Cogollos, R.; Rubio, J.M.; Villares, R.; Alarabe, A.; Cervera, A.; Lopez-Velez, R.	2010	To describe the cases of imported childhood malaria including clinical, epidemiological, laboratory, and diagnostic features of those who attended at a hospital in the southwest of Madrid.	45/46 (98%) of all immigrants with imported malaria acquired it in their African countries of origin, compared to the 2% of recent immigrants who came from Ecuador with imported malaria. All individuals in the VFR group acquired malaria in Equatorial Guinea (n=14) and 90% of all new immigrants acquired malaria in Equatorial Guinea (n=41)
[160]	Barnevik-Olsson, M.; Gillberg, C.; Fernell, E.	2010	To study the prevalence of autism in children of Somali background living in Stockholm	The high prevalence of autism in children of Somali origin in Stockholm reported in previous study was confirmed.
[191]	Lucas, M.; Nicol, P.; McKinnon, E.; Whidborne, R.; Lucas, A.; Thambiran, A.; et al	2010	To demonstrate the influence of age, ethnicity and clinical status on IGRA results and the limitations of using these tests in refugee children	For all three tests, the proportions of positive results were similar for children of African and Asian origin. T-SPOT.TB performed better in African children with other infections. For African children without identified co-morbid infections, neither assay was clearly superior in its likelihood to provide a definitive result. Positive agreement between the TST and both IGRAs was higher for African children
[145]	Agudelo-Suarez, A.A.; Ronda-Pérez, E.; Gil-González, D.; González-Zapata, L.I. Regidor E.	2009	To analyse the differences between low birth weight (LBW) and preterm birth (PB) according to the mother's nationality in newborns in Spain	In comparison with Spanish ones, the lowest risk of PB was observed in North African children (ORa= 0.77 $CI_{95\%}$ 0.74-0.80).
[45]	Agmon-Levin, N.; Elbirt, D.; Asher, I.; Torten, D.; Cohen, Y.; Gradestein, S.; et al	2009	The objective of the study was to investigate the HIV-mother-to-child transmission (MTCT) rate in Israel	The rate of HIV-MTCT in the entire cohort was 3.6% (11/300 pregnancies); eight infected infants were born to mothers of African origin. 86% of HIV infected mothers were of Ethiopian origin
[32]	Kouadio, I.K.; Koffi, A.K.; Attoh-Toure, H.; Kamigaki, T.; Oshitani, H.	2009	To describe the epidemiology of concurrent measles and rubella outbreaks in Côte d'Ivoire transit camps (TCs) for Liberian refugees during 2003–2004	From 19 January to 23 February 2004 (weeks 8–13), measles IgM testing showed that 61.1% were positive. The highest incidence rate (18.5%) of measles was observed in children aged <9 months. Rubella IgM testing revealed 74. 0% positive cases between 14 February and 25 April (weeks 11–21).
[36]	Cherian, S.; Forbes, D.; Sanfilippo, F.; Cook, A.; Burgner, D.	2009	To assess the association between H.pylori and helminth infection and	H. pylori infection was detected in 149/182 (81.9%). Children with H. pylori infection were older (mean

			growth in refugee children shortly after resettlement	8.5 years, standard deviation (SD) 4.2 years vs. 5.8 years, SD 4.5 years, p < 0.001). No gender differences were observed. Helminth infections were common (41.8%) but not associated with reduced growth or with H. pylori infection.
[157]	Sarafoglou, K.; Bentler, K.; Gaviglio, A.; Redlinger-Grosse, K.; Anderson, C.; McCann, M.; et al	2009	To report on an unusually high occurrence of profound biotinidase deficiency (BTD) within a single ethnic population and an overall high occurrence of combined partial and profound BTD in Minnesota	Of the 5 subjects with profound BTD, four of them were of Somali ethnic background. With four cases of profound BTD, the incidence among infants of Somali ethnic background is 1/1395. Of the 26 subjects identified through newborn screening as having partial BTD, none were of Somali ethnic background.
[46]	Åsander, A.S.; Björkman, A.; Belfrage, E.; Faxelid, E.	2009	To explore the frequency of HIV-infected African parents' disclosure of their status to their children and custody planning for their children's future to identify support needs among these families	In Sweden, the majority of HIV-infected parents are of African origin, and despite the introduction of anti-retroviral (ARV) medication, there has been no tendency for disclosures to children to increase. The fear of a double stigma—that of being both immigrant and HIV infected—is likely the main barrier to disclosure of HIV infection.
[81]	Renzaho, A.M.N; Swinburn, B.; Burns, C.	2008	To examine the association between acculturation and obesity and its risk factors among African migrant children in Australia	The further African children moved from traditional lifestyles, the less physically active they become and the more fatty foods they eat, which results in obesity.
[184]	Obeng, C.S.	2008	To examine dental health issues for African immigrant families of preschoolers living in the United States	The authors of this study found that 82% of the parents felt that it was unnecessary for a child to be taken to a dentist at an early age since their chances of contracting diseases at the dentist's office at that age were high. In addition, 48% of the parents had never taken their children to have their teeth checked by a dentist, and 61% of the parents reported that their children brushed their teeth once per day.
[151]	Gaspar de Matos, M.; Gaspar, T.; Simons-Morton, B.; Reis, M.; Ramiro, L.	2009	The aim of this study is to determine the influence of migrant status on sexual behavior and communication about "safer sex" and to identify ethnic-specific prevention issues	African adolescents tend to begin sexual life early, use condoms infrequently, and have more difficulties in talking with parents about sex. 2) Boys and girls agreed that it is up to the male to decide about condom use, and girls viewed even unprotected sex better than not being cared for. Some girls indicated that getting pregnant young was normative. 3) Both parents and adolescents expressed inhibition and lack of interest in talking to each other about sex and related issues. Sexuality and HIV were considered

				taboo themes for these parents and adolescents.
[35]	Cherian, S.; Forbes, D.; Sanfilippo, F.; Cook, A.; Burgner, D.	2008	To determine the prevalence and associated epidemiological features of Helicobacter pylori infection in child refugees in Western Australia	149 of these 182 (82%) had H. pylori infection. Age was an independent predictor of H. pylori infection. No sex differences were observed. Premigration antimalarial therapy (with sulfadoxine–pyrimethamine and artesunate) significantly reduced the prevalence of H. pylori infection (age-adjusted OR, 0.33; 95% CI, 0.15–0.75).
[34]	Cherian, S.; Forbes, D.A.; Cook, A.G.; Sanfilippo, F.M.; Kemna, E.H.; Swinkels, D.W.; Burgner, D.P.	2008	To investigate the relationships between hepcidin, cytokines, and iron status in a pediatric population with a high prevalence of both anemia and co-morbid infections	Gastro-intestinal infections (H. pylori and helminths) did not elevate urinary hepcidin or IL-6 levels in refugee children, nor were they associated with iron deficiency anemia.
[85]	Hintzpeter, B.; Scheidt-Nave, C.; Muller, M.J.; Schenk, L.; Mensink, G.B.	2008	To compare the prevalence of vitamin D deficiency between immigrants and nonimmigrants aged 1–17 y in Germany, taking into account the recommended vitamin D supplementation during infancy	Despite a significantly higher proportion of vitamin D supplement users and higher vitamin D intake index, children with an immigrant background were significantly more likely to have inadequate vitamin D concentrations.
[42]	McPherson, M.E.; Woodgyer, A.J.; Simpson, K.; Chong, A.H.	2008	To investigate a reported increase in tinea capitis in an English-language school to determine if it was an outbreak and whether control measures were warranted	Sudanese children accounted for 34.8% of cases, 44.4% of carriers, and 17.4% of children uninfected by tinea capitis. Other African children accounted for 8.7% of cases, 11.1% of carriers, and 5.8% of children were uninfected by tinea capitis.
[161]	Barnevik-Olsson, M.; Gillberg, C.; Fernell, E.	2008	To investigate the prevalence of autism in children with parents from Somalia living in Stockholm and to compare the prevalence in children of Somali background with that in the non-Somali group	The minimum prevalence of autistic disorder or pervasive developmental disorder not otherwise specified (PDDNOS) was 0.7% (17 of 2437; 95% confidence interval [CI] 0.37–1.03) among children with a Somali background compared to children with a non-Somali background; the corresponding prevalence was 0.19% (484 of 250 565; 95% CI 0.18–0.21; p<0.001).
[98]	Seal, A.; Kafwembe, E.; Kassim, I.A.; Hong, M.; Wesley, A.; Wood, J.; Abdalla, F.; van den Briel, T.	2008	To assess changes in the Fe and vitamin A status of the population of Nangweshi refugee camp associated with the introduction of maize meal fortification	The introduction of fortified maize meal led to a decrease in anaemia in children by 23.4% (P<0.001), and a decrease in vitamin A deficiency in adolescents by 26.1% (P<0.001).
[213]	Plotinsky, R.N.; Straetemans, M.; Wong, L.Y.; Brown, M.J.; Dignam, T.; Dana Flanders, W.; et al	2008	To identify risk factors for increasing lead levels among refugee children after resettlement in Manchester in 2004	Median age was 5.5 years at the time of second measurement of blood lead levels. Thirty-six (39%) of the refugee children had BLL2X10 mg/dL. Liberians and those born in refugee camps had higher geometric mean BLL2 than those not Liberian or not born in camps. Younger children and children with

				nutritional wasting before immigrating to the United States had a greater increase in geometric mean from BLL1 to BLL2, compared to older children and those without nutritional wasting.
[180]	Almerich-Silla, J.M.; Montiel-Company, J.M.; Ruiz-Miravet, A.	2008	To investigate the relationship between dental fluorosis and dental caries among western Saharan refugee children	The decayed, missing, or filled teeth (DMFT) score was 0.48 in the 6–7-yr-old children and 1.69 in the 11–13-yr-old children, with a caries prevalence (DMFT > 0 or decayed and filled primary teeth (dft) > 0) of 47.2 and 63.2%, respectively. Among the 6–7 yr-old children examined, 36.9% were free of fluorosis, 15.6% presented moderate fluorosis, and 7.8% presented severe fluorosis. Among 11–13 yr-old children, only 4.2% were free of fluorosis, 30.2% exhibited moderate fluorosis, and 27.4% presented severe fluorosis.
[30]	Nysse, L. J.; Pinsky, N.A.; Bratberg, J.P.; Babar-Weber, A. Y.; Samuel, T.T.; Krych, E.H.; et al	2007	To determine the seroprevalence of varicella antibody among recent Somali refugees living in Olmsted County, Minnesota, and to estimate the risk of varicella-zoster virus (VZV) infection in this group	Thirty-five subjects (18%) were seronegative for vari-cella. Males had a significantly higher seronegativity rate (25%) compared with females (12%); however, this association disappeared after adjustment for age and varicella infection history. Five percent (5/92) of adults were seronegative compared with 30% (30/101) of all children. Eight percent (5/61) of the adult females were seronegative, whereas none (0/31) of the adult males were seronegative.
[55]	Cherian, S.; Burgner, D.	2007	To assess the safety of selective admission of paediatric refugees with *Plasmodium falciparum* (Pf) malaria in Australia	Ninety paediatric refugees were infected with P falciparum, of whom 56 were treated as outpatients. Of the 34 children admitted to hospital, four had parasite loads >4%. Most children were treated with oral atovaquone-proguanil. Eighty-eight patients attended follow-up; all were compliant and none reported side-effects.
[118]	Stevens, G.W.; Vollebergh, W.A. M.; Pels, T.V.M.; Crijnen, A.A.M.	2007	To study the relation between acculturation and problem behavior in Moroccan adolescents	Girls with an ambivalent acculturation pattern showed more problems than the other girls on parent- and adolescent reported internalizing and externalizing behavior. For boys, no effects of acculturation on problem behavior were found. The high number of conflicts between parents and their ambivalently acculturated daughters partly explained the relation between acculturation and problem behavior.

[83]	Stellinga-Boelen, A.A.; Wiegersma, P.A.; Bijleveld, C. M.; Verkade, H.J.	2007	To assess the nutritional condition and growth of asylum seekers' children upon arrival and follow-up in the Netherlands.	The proportion of children who were obese/overweight increased in those seeking asylum in the Netherlands following their migration.
[86]	McGillivray, G.; Skull, S.A.; Davie, G.; Kofoed, S.E.; Frydenberg, A.; Rice, J.; Cooke, R.; Carapetis, J.R.	2007	To assess the pattern of and risk factors for vitamin D deficiency (VDD) in immigrant East African children living in Melbourne	Low 25-hydroxyvitamin D (25-OHD) levels (.50 nmol/L) occurred in 87% of children, and VDD (25-OHD .25 nmol/L) in 44%. Risk factors included age ,5 years, female gender, increased time in Australia, decreased daylight exposure and winter/spring season. Anaemia (20%), vitamin A deficiency (20%) ,and iron deficiency (19%) were also identified.
[120]	Goldblatt, H.; Rosenblum, S.	2007	To examine the experience of immigrant Jewish Ethiopian youth in Israel and its impact on their identity formation and determine which aspects contribute to the experience of being a Jewish Ethiopian youth in Israel.	Despite the stress and challenges of integration in Israeli society families are central to youth's sense of belonging. Schools need to establish definite disciplinary boundaries between adults and children for immigrant youths.
[134]	Harding, S.; Santana, P.; Cruickshank, J. K.; Boroujerdi, M.	2006	To investigate differences in birth weights between babies of foreign-born black African, Portugal-born black African, and Portugal-born white mothers	Compared with white Portuguese babies, mean birth weight of term babies of foreign-born African mothers was greater, and that of babies of Portugal-born African mothers was intermediate. Small preterm babies comprised 1.5% of white Portuguese babies, 2.3% of babies of Portugal-born African mothers, and 3.9% of babies of foreign-born African mothers ($p<0.05$ compared with white Portuguese babies). Compared with white Portuguese babies, mean birth weight of term babies, adjusted for sex, among Portugal-born African mothers was 24.6 g (95% CI, 70.1–20.9), and among foreign-born African mothers was +38.8 g (95% CI, 2.9–74.8).
[133]	Harding, S.; Boroujerdi, M.; Santana, P.; Cruickshank, J.	2006	To examine (i) whether the recently observed African–White differences in birth weights in Lisbon are replicated at a national level, (ii) temporal shifts in the birth weight distributions of babies of Portuguese and African mothers, and (iii) the factors influencing any changes in birth weights in these groups	Among African births there was an increase in births to teenage mothers and a decline to mothers from advantaged socioeconomic backgrounds. African (3297, SD 441 g) babies were not different but the percentage of small preterm births was higher among African (4.7%) than among Portuguese (2.9%) births. The left shift of the birth weight distributions was independent of maternal age, parity, and social factors among Portuguese babies, but among African babies the decrease appeared to be associated with socioeconomic advantage.

[79]	Hadley, C.; Sellen, D.	2006	To evaluate the usefulness and feasibility of methods to assess the prevalence of food insecurity and child hunger, and to examine associations between child hunger and measures of socioeconomic status and measures of acculturation in Liberian refugees and asylum seekers	85% of households were food insecure, and 42% experienced child hunger. Hunger was more likely to be indicated in households using foods stamps, with lower income, and lower education. Hunger was also more likely to be indicated in households where the primary shopper experienced difficulty shopping and with language.
[190]	Chemtob, D.; Weiler-Ravell, D.; Leventhal, A.; Bibi, H.	2006	To analyze the specific impact of our screening procedures on active TB among children in Israel	Most cases (81.8%) were foreign born, predominantly (88.2%) immigrants from Ethiopia and, therefore, huge differences existed in TB incidence rates according to countries of origin. TB was more frequently diagnosed among young children (aged 0–4 years) of Ethiopian origin (39.9%) compared to those from the former Soviet Union (17.5%) (P = 0.067). TB was diagnosed within 3 years of arrival most (83.6%) of the Ethiopian children.
[82]	Renzaho, A.M.; Gibbons, C.; Swinburn, B.; Jolley, D.; Burns, C.	2006	To assess the anthropometric status of 337 sub-Saharan African children aged between 3-12 years who migrated to Australia	Obesity and overweight are very prevalent in SSA migrant children and undernutrition, especially wasting, was also not uncommon in this target group. The prevalence rates for overweight and obesity were 18.4% (95%CI: 14 - 23%) and 8.6% (95%CI: 6% -12%) respectively. The prevalence rates for the indicators of undernutrition were: wasting 4.3% (95%CI: 1.6%-9.1%), underweight 1.2% (95%CI: 0.3%-3.0%), and stunting 0.3 (95%CI: 0.0%-1.6%).
[57]	Lavoie, P.M.; Carceller, A.; Robert, M.; Lebel, M.H.; Moore, D. L.; Rashed, S.	2006	To review the clinical features and risk factors for complications and treatment of malaria in a pediatric cohort mainly composed of immigrant children	Of the 94 children with malaria from Africa, 80% had P. falciparum, 11% P. ovale, 5% P. malariae, 4% P.vivax. P. falciparum affected a larger proportion of children from Africa relative to other immigrant regions of origin. Of the 121 cases analyzed, 93 of the patients originated from Africa. Ten children with serious complications had P. falciparum infection (P = 0.03), and presented within one month of arrival from their respective endemic country. All of these patients arrived from Africa.
[93]	Seal, A.J.; Creeke, P.I.; Mirghani, Z.; Abdalla, F.; McBurney, R.P.; Pratt, L.S.; Brookes, D.; Ruth, L.J.; Marchand, E.	2005	To assess the level of iron deficiency anemia and vitamin A deficiency in populations dependent on long-term international food aid and humanitarian assistance	The research illustrates evidence of high level of micronutrient malnutrition (iron and vit A) and moderate-to-high levels of protein energy malnutrition were seen in several of the camps.

[148]	Johnson, E.B.; Reed, S.D.; Hitti, J.; Batra, M.	2005	To compare maternal and neonatal morbidity among Somali immigrants, US-born blacks and whites in Washington state	Somali women more commonly had caesarean deliveries associated with fetal distress and failed induction of labor. They were 9 times more likely than both control groups to deliver after 42 weeks gestation, and 4 times more likely than US-born black women and 8 times more likely than white women to have oligohydramnios. Newborns of Somali women were at increased risk for prolonged hospitalization, lower 5-minute Apgar scores, assisted ventilation, and meconium aspiration.
[211]	Cooke, R.; Murray, S.; Carapetis, J.; Rice, J.; Mulholland, N.; Skull, S.	2004	To analyze the demographics and utilization of health services by pediatric refugees from East Africa and to assess the implications for service planning and provision	Availability of interpreters and information on health services were the main factors hindering access to care. These data have informed future service planning at the Clinic. Ongoing data collection is key to maintaining a responsive, targeted service for a continually changing population.
[139]	Hargreaves, J.R.; Collinson, M.A.; Kahn, K.; Clark, S. J.; Tollman, S.M.	2004	To investigate differentials in mortality between children from former Mozambican refugee and host South African households in a rural sub-district in the north-east of South Africa	This study demonstrates higher childhood, but not infant, mortality rates among children from former Mozambican refugee households compared with those from host South African households in rural South Africa. The lack of legal status and lower wealth of many former Mozambican refugees may partly explain this disparity.
[174]	Cote, S.; Geltman, P.; Nunn, M.; Lituri, K.; Henshaw, M.; Garcia, Raul I.	2004	To describe the prevalence of caries experience and untreated decay among newly arrived refugee children stratified by their region of origin and compared with US children	African refugee children had significantly lower dental caries experience as well as fewer untreated caries as compared with similarly aged Eastern European refugee children. They were also less likely to have ever been to a dentist. Possible reasons for these findings may include differences in exposure to natural fluoride in the drinking water, dietary differences, access to professional care, and cultural beliefs and practices.
[56]	Depoortere, E.; Guthmann, J.P.; Sipilanyambe, N.; Nkandu, E.; Fermon, F.; Balkan, S.; Legros, D.	2004	To measure patient adherence to Artemisinin-based combination therapy (ACT) in children up to age 5 years under routine conditions of prescription in a refugee settlement	We found 21.2% of the patients to be certainly non-adherent, 39.4% probably non-adherent, and 39.4% (95% CI [31.6–47.6]) probably adherent. Insufficient explanation by the dispenser was identified as an important reason for non-adherence.
[147]	Vahratian, A.; Buekens, P.; Delvaux, T.; Boutsen, M.; Wang, Y.; Kupper, L.L.	2004	To compare birthweights and frequencies of preterm birth for North African and Belgian infants	Infants of North African immigrants were heavier at birth and were less likely to be born low birthweight or preterm, compared to infants of Belgian women. North African

				women were more likely to live with a partner while pregnant. After adjusting for sociodemographic and maternal factors, the estimated difference in mean birth weight between North African and Belgian women was about 74 g (p=0.05) among all births.
[44]	Hallgren, J.; Petrini, B.; Wahlgren, C.F.	2004	To describe the mycological and clinical data in children diagnosed with tinea capitis in a hospital setting in Stockholm	The study showed a striking predominance of scalp infection caused by T. violaceum among children of eastern African extraction. Why children of African extraction seem to be at higher risk of infection with these fungi than others remains unclear. Children of immigrants tend to be infected with dermatophyte species common in their country of origin, irrespective of how long they have lived in their adopted country.
[159]	Kamer, A.; Zohar, A.H.; Youngmann, R.; Diamond, G.W.; Inbar, D.; Senecky, Y.	2004	To estimate the prevalence of pervasive developmental disorder (PDD) among immigrants to Israel and Israeli natives	Among immigrants, the rate of PDD in Ethiopian-born children was lower than that of those born in other countries. The rate of PDD in immigrant Ethiopian children was much lower than in native Israeli children of Ethiopian extraction.
[97]	Lopriore, C.; Guidoum, Y.; Briend, A.; Branca, F.	2004	To assess the effect of a highly nutrient-dense spread fortified with vitamins and minerals, with or without antiparasitic metronidazole treatment, in correcting retarded linear growth and reducing anemia in stunted children	Fortified spread (FS), and not unfortified spread (US), induces catch-up growth in stunted children whose diets are poor in micronutrients. Our trial provides support for delivering multiple micronutrients to reverse stunting and reduce anemia in children up to age 6 years. Linear growth of children fed FS was 30% faster at 3 months than in US and control groups, after which height-for-age z scores increased only slightly in the Increase in hemoglobin concentrations in the FS group at 6 mo was twofold that in the US and control groups (37 ± 40, 19±15, and 16±17 g/L, respectively; P<0.0001), and anemia was reduced by nearly 90%.
[29]	Javaloy, J.; Ferrer, C.; Vidal, M.T.; Alio, J.L.	2003	To ascertain the prevalence of acute trachoma as a supposed endemic disease among infants in the Saharan refugee camps of Tindouf (Algeria) and to evaluate the efficacy of treatment with a single dose of azithromycin	Chlamydia trachomatis was suspected in 2.47% of the children, papillary hypertrophy being the most frequently seen clinical sign. Scarring changes secondary to trachoma were detected in 11.7% of the children. Only four cases (0.8%) were positive to the immunoassay test and 12 cases (2.27%) were positive by polymerase chain reaction (PCR). After treatment a second PCR was performed on

				positive children and they were negative of chlamydia DNA amplification.
[130]	Stevens, G.W.; Pels, T.; Bengi-Arslan, L.; Verhulst, F.C.; Vollebergh, W.A.; Crijnen, A.A.	2003	To compare emotional and behavioral problems of Moroccan immigrant children to those of Dutch native children and Turkish immigrant children	Moroccan parents reported as many problems as Dutch parents, but less problems than Turkish parents. Teachers stated substantially more externalizing problems were reported for Moroccan pupils compared to Dutch and Turkish pupils. Moroccan adolescents themselves reported less problems than Dutch and Turkish adolescents.
[185]	Vered, Y.; Schwarts, N.; Mann, J.; Sgan-Cohen, H.D.	2003	To investigate periodontal status and oral hygiene practices among recent new immigrants from Quara, Ethiopia the aim to maintain and promote their periodontal health	55.7% of 13- to 17-year-olds had a community periodontal index score of 2 indicating calculus.
[101]	Renzaho, A.; Renzaho, C.	2003	To evaluate the public health and nutritional situation of refugee children in Katale camp, Eastern Zaire, after two years of nutritional and health intervention from 1994 to 1996	Public health interventions in Katale camp 1994 to 1996 reduced mortality and morbidity rates dramatically. This was not reflected in the malnutrition rates for children under five years, which remained stable after an initial fall despite two years of nutritional intervention.
[91]	Reifen, R.; Haftel, L.; Manor, G.; Sklan, D.; Edris, M.; Khoshoo, V.; Ghebremeskel, K.	2003	To determine the growth patterns of immigrant children after changes in their nutritional habits	The 7- to 11-y-old Israeli-born boys were heavier ($P<0.03$) and had higher mean arm muscle circumference transthyretin, and magnesium ($P<0.0001$) and lower albumin ($P<0.0001$) and triceps skinfold thickness ($P<0.005$) than did the corresponding caravan-dwelling boys. Israeli girls were heavier ($P<0.006$) and had higher wt/ht ratio ($P<0.02$), MAMC, transthyretin, calcium, and magnesium ($P<0.0001$) and lower albumin ($P<0.05$) and TSF ($P<0.0001$) than did the Ethiopian-born girls of the same age range. There was a significant difference in weight, wt/ht ratio, TFS, MAMC, transthyretin, calcium, and magnesium ($P<0.0001$) between the Ethiopian- and Israeli-born boarding schoolboys.
[63]	Rice, J.E.; Skull, S.A.; Pearce, C.; Mulholland, N.; Davie, G.; Carapetis, J.R.	2003	To describe the prevalence and risk factors for intestinal parasite carriage among children recently arrived from East African countries	Intestinal parasites are common among East African immigrant children.
[94]	Tumwine, J.K.; Barugahare, A.A.	2002	To establish magnitude and risk factors for Malnutrition in Kasese district at the Uganda-congo border	Half of the surveyed children were stunted. Risk factors for stunting include not consuming milk, low literacy of the father, having no immunization card, and children age 12 to 23 months not breastfeeding.

[197]	St Sauver, J.L.; Jacobson, R.M.; Vierkant, R.A.; Jacobsen, S.J.; Green, E.M.; Poland, G.A.	2002	To compare measles, mumps, and rubella antibody levels in Somali immigrant, Hispanic migrant, and US children in Rochester, MN, and to determine whether parental vaccination reports predict seropositivity	Somali children reported vaccination at significantly older ages compared with Hispanic or Rochester children (P<.001). None of the Somali vaccinations were recorded in a written medical record, but 25 (37%) of 68 measles vaccinations and 24 (36%) of 67 mumps and rubella vaccinations were recorded in a written personal file. Hispanic children were slightly more likely to have positive measles antibody levels compared with the Somali children (pairwise P=.07). Among the 9 Somali children who did not receive measles vaccination (or were unsure if they had been vaccinated), 8 (89%) were seropositive for measles antibodies. Parental vaccination reports had high positive predictive values (71-100%) and low negative predictive values (13-25%).
[28]	Huerga, H.; Lopez-Velez, R.	2002	To ascertain a morbidity profile for infectious diseases in sub Saharan African immigrant children evaluated in a reference tropical medicine unit	79% of children had infectious pathology, and 33.3% (33 cases) were infected by 3 or more infectious diseases (range, 3-5). High number of infectious diseases was diagnosed in sub-Saharan African immigrant children including malaria, parasitic infections, TB, and viral hepatitis.
[152]	Essen, B.; Bodker, B.; Sjoberg, N.O.; Gudmundsson, S.; Ostergren, P. O.; Langhoff-Roos, J.	2002	To examinate the high rate of perinatal mortality among children of immigrant women from the Horn of Africa raises the question of whether there is an association between female circumcision and perinatal death	There was no evidence that female circumcision of the mother was related to perinatal death.
[58]	Williams, J.P.; Chitre, M.; Sharland, M.	2002	To identify changes in the presenting number and species of imported malaria in children in southwest London	Imported childhood P. falciparum malaria is increasing in southwest London associated with increasing travel from sub-Saharan Africa. Over the 25-year period there has been no improvement in chemoprophylaxis rates or time to diagnosis.
[38]	Lamb, S.R.; Rademaker, M.	2001	Not clearly stated	Among the 51 patients with isolates of *T. violaceum* (TV) or *T. soudanense* (TS) on scalp sites, 46 were Somalian children. Of the 46 Somali children with scalp isolates, only 4 had TS and 42 had TV. Only 4 children were cured, and the remainder did not have the outcome available. Of the 9 cases with TV or TS isolates from non-scalp sites, 4 isolates derived from Somali children. Of these 4 children, 3 had

				TV and one had TS. Outcomes of the infection were not reported.
[215]	McMahon, C.; Callaghan, C.O.; O'Brien, D.; Smith, O.P.	2001	To determine the prevalence of childhood SCD in Ireland and to predict requirements for a comprehensive care and assessment programme	All 20 children with SCD were from Africa (12 from Nigeria, 4 from Congo, 3 from Angola and one from Zaire). Their Hb level was 6.4-9.7 g/dL with a median of 7.7 g/dL and all consistently fail to express the Duffy red cell antigen (Fya/Fyb).
[61]	Geltman, P.L.; Radin, M.; Zhang, Z.; Cochran, J.; Meyers, A.F.	2001	To analyze growth and identify related medical conditions among refugee children in Massachusetts	Of the African refugee children screened for ova and parasites, 50% tested positive. Infection with pathogenic parasites was associated with increased risk of low height-for-age z scores (OR=2.4; 95% CI=1.6-3.4). Of African refugee children screened for TB via the purified protein derivative (PPD) tuberculin skin test, 25% tested positive. A positive PPD result was associated with lowered likelihood of having height-for-age z scores less than −2 (OR=0.6; 95% CI=0.3, 0.9). Of the African refugees who were screened for dental abnormalities, 72% tested positive.
[74]	Ratsch, I.M.; Catassi, C.	2001	To characterize the clinical and nutritional impact of coeliac disease (gluten-sensitive enteropathy) among Saharawi children living as refugees in Algeria	Celiac disease has a negative effect on the health status of Saharawi refugee children. Because of the high prevalence of the condition in the Saharawi, a specific programme for treating all affected individuals should be established. Further studies are required to quantify the impact of celiac disease in other areas of the developing world.
[136]	Jamieson, D.J.; Meikle, S.F.; Hillis, S.D.; Mtsuko, D.; Mawji, S.; Duerr, A.	2000	To estimate incidence of and determine risk factors for poor pregnancy outcomes and to calculate the contribution of mortality from neonatal and maternal deaths to overall mortality in a refugee camp	The fetal death rate was 45.6 per 1000 births, the neonatal mortality rate was 29.3 per 1000 live births, and 22.4% of all live births were low birth weight. Compared with women without poor pregnancy outcomes, those with poor pregnancy outcomes were more likely to report prior high socioeconomic status and having 3 or more episodes of malaria during pregnancy.
[140]	Essen, B.; Hanson, B.S.; Ostergren, P.O.; Lindquist, P. G.; Gudmundsson, S.	2000	To investigate whether the maternal country of origin affected the risk for perinatal mortality and to determine its relationship to risk factors	Women of foreign origin, especially from sub-Saharan Africa, have a higher risk of perinatal mortality than native Swedish women. The differences in mortality could not be explained by risk factors. The results suggest that women and newborns from sub-Saharan Africa should be given more intense surveillance on all levels of perinatal care in order to reduce perinatal mortality.

[127]	Berman, H.	1999	To explore how two groups of children who grew up amid violence "make sense" of their experience	Themes of normalcy, peace, and contentment, prior to war, were common amongst refugee children. They believed that their lives would remain that way forever. As they stated repeatedly, there was no reason to believe otherwise. None of the refugee children had any preparation for the changes which ensued. Although they had only vague understandings as to what the fighting was about or who the warring factions were, they had very clear memories as to the actual events, what they saw and what they heard.
[106]	Peltzer, K.	1999	To determine the extent of trauma and mental health problems	The traumatic event in all the 56 children happened more than six months previously. Twelve children (20%) were suffering from chronic PTSD; their age range was from six to 14 years with a mean of 11.2 years (Q1= 8.3; Q,= 12.9).
[105]	Paardekooper, B.; de Jong, J.T.; Hermanns, J.M.	1999	To study the psychosocial effects of the war situation and subsequent flight on South Sudanese children who were compared to a group of Ugandan children who did not have these experiences of war and flight	Compared to Ugandan children, the Sudanese reported significantly more PTSD-like complaints, behavioural problems, and depressive symptoms. The many psychological complaints from the Sudanese children could be caused by traumatic events they experienced as well as by their difficult daily circumstances.
[132]	Zlot, A.	1999	To address body image among Ethiopian-Israeli teenage girls	Ethiopian-Israeli girls found a balance between adopting Western concepts of beauty and preserving their Ethiopian traditions. Ethiopian-Israeli girls did not differentiate between the body, mind, and sprit; the girls maintained a holistic perception of the body. Different body parts were also assigned symbolic meaning.
[207]	Nudelman, A.	1999	To explore to what extent traditional Ethiopian culture influences health practices of immigrant adolescents, in what circumstances are they more likely to adopt Western patterns of response as reflected by those of the Israeli culture.	Several situations of illness were presented, with an analysis of the differential behaviour patterns of Ethiopian immigrant pupils in Israeli youth villages. A similar process was evident among the different cases. In the first stage, there was a tendency to act according to the accepted mode of behaviour in Israel, and to go to the clinic for medical treatment.
[171]	Roville-Sausse, F.	1998	To determine the influence of ethnic, cultural, and socioeconomic differences on the growth of children, particularly in the younger preschool years	After the first year of life, the means for the BMI of the children born to sub Saharan immigrant parents are lower than means for French children. This suggests poorer

				nutritional status, which may be associated with the marginal socioeconomic status of African families living in the Paris area.
[146]	Buekens, P.; Masuy-Stroobant, G.; Delvaux, T.	1998	To analyze all available years of vital records to compare birth weights of newborns of North African and Belgian origin	Despite their low socioeconomic status, North African immigrants have high birth weights.
[41]	Cuetara, M.S.; Del Palacio, A.; Pereiro, M.; Noriega, A.R.	1998	To determine how many cases of tinea capitis (TC) were present and to assess the carriage rate of dermatophytes in the scalps of clinically normal school children	Of the 52 (0.52%) children with dermatophytes in the scalp, 12 of these cases were in children from Africa. Of these 12, 10 children had TC.
[40]	Cuetara, M.S.; del Palacio, A.; Pereiro, M.; Amor, E.; Alvarez, C.; Noriega, A.R.	1997	To detect dermatophytes on the scalp was undertaken in 5000 unselected school children aged between 3 and 16 years	There was a significantly higher proportion of cases of tinea capitis (P< 0.001) (particularly due to Trichophyton tonsurans, P< 0.001) and of cases of asymptomatic scalp carriers (P< 0.05 (particularly due to Trichojdyton tonsurans, P< 0.001) in the immigrant population of African origin.
[202]	Vos, C.; Reeser, H.M.; Hirasing, R.A.; Bruining, G.J.	1997	To assess the incidence of type 1 diabetes in under 20 years of age in the Netherlands, among Moroccan children in the Netherlands, correcting for determining the incidence in the period 1988–1990	In all age categories, the incidence of type 1 diabetes is higher in Moroccan children than in the Dutch children.
[31]	Swerdlow, D.L.; Malenga, G.; Begkoyian, G.; Nyangulu, D.; Toole, M.; Waldman, R.J.; Puhr, D.N.; Tauxe, R.V.	1997	To report an epidemiologic investigation of this cholera outbreak and document the challenges of providing adequate treatment and prevention in these circumstances	Of the 173 patients whose records were reviewed following admission to the intravenous tent, 28% of patients were <6 years old. Twenty-four (60%) of the 40 persons whose deaths were reviewed were children under 4 years of age, compared to 29 (17%) of the 173 selected patients who were children (RR=4.5, [CI] 2.6–7.9). Fever was especially common among children.
[54]	Wolday, D.; Kibreab, T.; Bukenya, D.; Hodes, R.	1995	To assess the susceptibility in vivo of P. falciparum to chloroquine and pyrimethamine-sulfadoxine in Goma refugee camp in Zaire.	Children with malnutrition were more likely to have higher parasite densities and slower clearance of parasites than children without malnutrition and the prevalence and degree of resistance in vivo were more pronounced in malnourished children. In the chloroquine treatment group, 62% of all P. falciparum infections among normally nourished children were resistant in vivo, compared to 77% of infections among the malnourished children. In those treated with pyrimethamine-sulfadoxine, 58% of all infections in normal children were resistant, compared to 86% of the infections in children with malnutrition.

[49]	Irova, T.I.; Burtonboy, G.; Ninane, J.	1995	To report experience of "vertically"-acquired pediatric HIV infection, the mother- to-child transmission rate, and the clinical spectrum of HIV infection in our population of children, most of whose families came from Central Africa	The findings are consistent with other studies from Africa and present evidence for a higher mother-to-infants transmission rate of HIV-1 in children born to HIV+ mothers in Africa than in industrialized countries.
[129]	Ravid, C.; Spitzer, A.; Tamir, B.; Granot, M.; Noam, R.	1995	To examine the internal body perceptions among Ethiopian Jews who emigrated to Israel	Ethiopian children, unlike western children, did not think of the body in terms of different biophysical functions. Rather they perceived it as a holistic system in which a divine providence was responsible for organs working together harmoniously.
[206]	Luboshitzky, R.; Dgani, Y.; Atar, S.; Qupty, G.; Tamir, A.; Flatau, E.	1995	To assess the prevalence of goiter and thyroid dysfunction in a population of 534 Ethiopian children, one year after arrival in Israel.	The high prevalence of goiter in this group of Ethiopian children with the low frequency of hypothyroidism may be attributed to the combined effects of food goitrogens and iodine deficiency prevailing in Ethiopia. The standard Israeli diet seems to be adequate in respect to iodine requirements, and no iodine enrichment is needed for children immigrating from Ethiopia.
[90]	Bern, C.; Nathanail, L.	1994	To identify children in need of supplementary feeding based on low weight-for-height (to assess the efficiency of MUAC (mid upper arm circumference) tool in measuring malnutrition in children of the refugee camps)	Among 3681 refugee children measured, 12% had low weight-for-height. Those approximately 6-11 months of age had the highest proportion of malnourishment, based on mid upper arm circumference (MUAC) . This measure is not accurate in measuring malnutrition in children, rather weight for height surveys are a more efficient screening tool.
[27]	Faustini, A.; Franco, E.; Saitto, C.; Cauletti, M.; Zaratti, L.; Papini, P.; Ali' Ahmed, S.; Zampieri, F.; Ierussi, A.; Panna, A.; Perucci, C.A.	1994	To evaluate the prevalence of HAV, HBV, HCV and HDV infections and to assess their possible risk factors.	HAV prevalence of 87.5% prevalence in children under 12. No subject under 11 was HBV positive. No children were HCV positive.
[173]	Holan, G.; Mamber, E.	1994	To assess the prevalence of primary canine tooth bud extractions in a group of Ethiopian Jewish children in Israel and to report on dental abnormalities associated with these early extractions	Forty-six (74%) mandibular compared to only 16 (26%) maxillary canines had been extracted. Another 19 primary canines had hypoplastic defects, probably the result of unsuccessful extractions. Associated dental abnormalities included hypoplasia of the permanent successors and adjacent primary and permanent teeth, displacement of permanent teeth, midline shift to the extraction side, missing primary lateral incisors and distal eruption of permanent lateral incisors.

[64]	Mumcuoglu, K. Y.; Miller, J.; Manor, O.; Ben-Yshai, F.; Klaus, S.	1993	To screen a representative number of Ethiopian immigrants for ectoparasites a few days after their arrival in Israel	The infestation rate of head lice among Ethiopian children varied between 65-100% in the various age-groups compared to Israeli children whose infestation rate ranged between 0 and 18%. Children 6-11 years old were the most infested group and no differences between girls and boys were found. Among Ethiopians, more children than adults were infested with head lice ($P<0.05$).
[177]	Sgan-Cohen, H. D.; Steinberg, D.; Zusman, S.P.; Sela, M.N.	1992	To determine the prevalence of dental caries, fluoride supplementation, and fluorosis, and to establish the dietary patterns, the microbial content of saliva, salivary flow rates, and pH levels in recent immigrants from rural Ethiopia	Salivary pH levels were generally similar between the Ethiopian group and the controls. Salivary flow was significantly higher for the Ethiopians (1.93 mL/min) than for controls (1.16 mL/min). Low levels of caries in this population can be attributed to an almost sugar-free diet and high salivary flow, but not to the composition of oral microflora.
[196]	Porter, J.D.; Gastellu-Etchegorry, M.; Navarre, I.; Lungu, G.; Moren, A.	1990	To review the measles surveillance in the camps, discuss the reason for outbreaks, and identify continued need for an effective single dose measles vaccine for children less than nine months of age	Children were being inappropriately vaccinated, either being vaccinated at less than six months of age (2-29%), or failing to receive a second dose if vaccinated at six months (0-25%). Measles outbreaks again indicate the need to improve vaccine coverage with the existing Schwarz vaccine, and also highlight the urgent need for an effective single dose measles vaccine for children less than nine months of age.
[176]	Sarnat, H.; Cohen, S.; Gat, H.	1987	To measure the changing pattern of dental caries, periodontal health status, and tooth cleaning behaviour among a cohort of Ethiopian immigrants to Israel between the years 1999–2005	The results showed a nearly sixfold difference in caries prevalence between the Ethiopian and the Israeli group (1.5/9.6). No differences could be seen clinically between the Ethiopians who immigrated more than 1 year ago and those who arrived within the last year. Radiographically, a significant difference was found between the two Ethiopian subgroups. Those who arrived more than 1 year ago had nearly three times more initial caries. Oral hygiene was poorer in the Ethiopian group than in the Israeli group; little difference was found in gingival health.
[144]	Yudkin, P.L.; Harlap, S.; Baras, M.	1983	To examine a group of births in West Jerusalem in 1975-1976 and to test whether ethnic group differences in birthweight were then apparent and, if so, whether they could be accounted for by differences in maternal body size or by other social or demographic factors	Birthweight was higher if the mother had immigrated to Israel after the age of 10 years than if she had immigrated at a younger age, or had been born in Israel.

[99]	Taylor, W.R.	1983	To feed the undernourished and protect the nutritionally vulnerable groups by providing them with supplementary food and to establish procedures for continuous surveillance of nutritional status of individuals and population groups	Out of 1006 children who attended at least one of two daily sessions, 53% exceeded the discharge level and 26% were in the category 80%-84% of the reference median weight for height. The prevalence of acute malnutrition in the camp population at Daray Ma'an was 34.7 to 35.3%. The small intracluster variation in the sample indicates that acute malnutrition was distributed uniformly throughout the camp.
[158]	Goel, K.M.; Thomson, R.B.; Sweet, E.M.; Halliday, S.	1981	To assess growth in relation to country of birth, time of parental residence in Scotland, child order in family, social class, living conditions, and dietary factors	For standardised height against age, differences in height for ethnic groups was consistent over all ages. No significant differences were noted between boys and girls of any ethnic group. For mean standardised height analysed by ethnic group, the mean for the African group was significantly higher (p<O.Ol) than the mean for the Asian group. For mean bone age for ethnic group, the African mean was significantly higher (p<0.01) than other group means. For weight according to Tuxford’s Index analysed by ethnic group (children 0-3 years), Africans had the largest mean and was significantly higher compared to Scottish, Asian, and Chinese children (p<0.01).
[60]	Goel, K.M.; Logan, R.W.; House, F.; Connell, M.D.; Strevens, E.; Watson, W.H.; Bulloch, C.B.	1978	Children were screened for abnormal haemoglobins, haemoglobin values, serum iron, serum transferrace, and red cell folate concentrations	The most frequently occurring abnormality was the sickle cell trait in African children and the next commonest was the Beta-Thalassaemia trait in Asian and Chinese children. No purely Scottish child was found to have a haemoglobinopathy. Findings suggest the need for a hemoglobinopathy clinic as essential part of the routine paediatric service in the UK.
[53]	Asch, A.J.	1976	To describe the epidemiology of malaria diagnosed at a tertiary care pediatric center in the multicultural city of Toronto	Of the *P. falciparum* infections, 32% were from Ghana, 28% were from Nigeria, and 7% were from Ivory Coast - the top 3 countries of origin for people infected by *P. falciparum* are all African countries. None of the top 3 countries of origin for *P. vivax* are African countries. One case of 2% paracitenia was identified in an 8-year-old girl from Nigeria.
[59]	Thompson, R.G.; Hutchison, J.G.; Johnston, N.M.	1972	Not clearly stated	The Kenyan population comprised roughly equal numbers of boys and girls with ages between 4 and 17 years, and their carriage of helminths was similar. These children have a

				much lower carriage rate (16%) than the Asian or American populations, and hookworm, hymenolepis, ascaris, and trichuris each contribute a substantial percentage to the total. The Ugandans show an even lower carriage rate (7%) than the Kenyans and due to this as well as the small size of the population examined (67 children), some species of worms are probably not represented.
[142]	Legg, S.; Davies, A.M.; Prywes, R.; Sterk, V.V.; Weiskopf, P.	1970	To analyze some demographic and socioethnic attributes of women who delivered underweight babies in Jerusalem in 1964-67	Between the different ethnic groups of Jews, the crude rate of low birth weight was lowest for North African children. When adjusted for differences in maternal age, birth order, and education, North African children faced a significant deficiency of low birth weight. In North African infants, maternal age had no significant effect on low birth weight. In the Asian and North African immigrant mothers, education is of low significance in contrast to the Israeli-born and western immigrant groups.
[212]	Bennett, F.J.; Jelliffe, D.B.	1965	To determine the possibility of eliciting exactly what health services immigrant mothers made use of for their babies and to determine patterns of usage of services to the health of the baby and urbanization status of the parents	20 of the 60 children were Child Welfare Clinic attendees. 13 of the 57 children (with an adequate history) were taken to Mulago hospital during this period and seven to the Catholic hospital. 27 babies had medicine bought for them, usually from the nearest shop but occasionally from a chemist. Many children received treatment in a variety of places often depending on the severity of the illness and financial resources/permission from the father. Reasons for hospital attendance seemed often to be the mothers' estimation of the seriousness of the illness (determined by the child's temperature and crying). Proximity of the clinic was the most important factor in determining its use.
